# Temporal manipulation of the *Scn1a* gene reveals its essential role in adult brain function

**DOI:** 10.1093/brain/awad350

**Published:** 2023-10-10

**Authors:** Claudia Di Berardino, Martina Mainardi, Simone Brusco, Elena Benvenuto, Vania Broccoli, Gaia Colasante

**Affiliations:** Stem Cell and Neurogenesis Unit, Division of Neuroscience, IRCCS San Raffaele Scientific Institute, 20132 Milan, Italy; Stem Cell and Neurogenesis Unit, Division of Neuroscience, IRCCS San Raffaele Scientific Institute, 20132 Milan, Italy; Stem Cell and Neurogenesis Unit, Division of Neuroscience, IRCCS San Raffaele Scientific Institute, 20132 Milan, Italy; National Research Council (CNR), Institute of Neuroscience, 20129 Milan, Italy; Stem Cell and Neurogenesis Unit, Division of Neuroscience, IRCCS San Raffaele Scientific Institute, 20132 Milan, Italy; Gene and Cell Therapy PhD Program, Vita- Salute San Raffaele University, 20132 Milan, Italy; Stem Cell and Neurogenesis Unit, Division of Neuroscience, IRCCS San Raffaele Scientific Institute, 20132 Milan, Italy; National Research Council (CNR), Institute of Neuroscience, 20129 Milan, Italy; Stem Cell and Neurogenesis Unit, Division of Neuroscience, IRCCS San Raffaele Scientific Institute, 20132 Milan, Italy

**Keywords:** Dravet syndrome, epilepsy, behavioural alterations, autistic features

## Abstract

Dravet syndrome is a severe epileptic encephalopathy, characterized by drug-resistant epilepsy, severe cognitive and behavioural deficits, with increased risk of sudden unexpected death (SUDEP). It is caused by haploinsufficiency of *SCN1A* gene encoding for the α-subunit of the voltage-gated sodium channel Na_v_1.1. Therapeutic approaches aiming to upregulate the healthy copy of *SCN1A* gene to restore its normal expression levels are being developed. However, whether *Scn1a* gene function is required only during a specific developmental time-window or, alternatively, if its physiological expression is necessary in adulthood is untested up to now.

We induced *Scn1a* gene haploinsufficiency at two ages spanning postnatal brain development (P30 and P60) and compared the phenotypes of those mice to *Scn1a* perinatally induced mice (P2), recapitulating all deficits of Dravet mice.

Induction of heterozygous Na_v_1.1 mutation at P30 and P60 elicited susceptibility to the development of both spontaneous and hyperthermia-induced seizures and SUDEP rates comparable to P2-induced mice, with symptom onset accompanied by the characteristic GABAergic interneuron dysfunction. Finally, delayed *Scn1a* haploinsufficiency induction provoked hyperactivity, anxiety and social attitude impairment at levels comparable to age matched P2-induced mice, while it was associated with a better cognitive performance, with P60-induced mice behaving like the control group.

Our data show that maintenance of physiological levels of Na_v_1.1 during brain development is not sufficient to prevent Dravet symptoms and that long-lasting restoration of *Scn1a* gene expression would be required to grant optimal clinical benefit in patients with Dravet syndrome.

## Teaser

Di Berardino, Mainardi, Brusco *et al.* show that induction of *Scn1a* haploinsufficiency in mice at postnatal day (P)2, P30 or P60 elicits a classic Dravet syndrome phenotype. Seizure severity, mortality rate and behavioural alterations are comparable in all three groups, but cognitive performance is better in P30 and P60-induced mice.

## Introduction

Dravet syndrome is a severe infantile epileptic encephalopathy, characterized by hyperthermia-induced seizures that evolve in drug-resistant epilepsy and relevant cognitive and behavioural deficits.^1^ Patients also face an increased risk of sudden unexpected death in epilepsy (SUDEP), estimated to be 15-fold greater than in other childhood-onset epilepsies. In most of the patients, haploinsufficiency of the *SCN1A* gene, due to heterozygous missense or nonsense mutations, is the underlying genetic cause of this disease.^2^ The *SCN1A* gene encodes for the α-subunit of the voltage-gated sodium channel Na_v_1.1. Current pharmacological treatments for Dravet syndrome,^3-6^ although largely improved and optimized in comparison to the past years, are ineffective in completely controlling convulsive attacks or delaying subsequent neurological symptoms. Therefore, a number of gene-based therapeutic strategies are in development.^7-9^

We have previously reported that *Scn1a* gene reactivation after symptom onset in a Dravet genetic background can recover most symptoms,^10^ indicating that efficient gene/genetic therapies aiming at re-establishing physiological levels of Na_v_1.1 in different brain regions can be effective as a cure.

However, from a therapeutic perspective, it would be important to determine whether *Scn1a* is also required after the critical developmental time-window or whether ensuring physiological levels of Na_v_1.1 during early postnatal life is sufficient to prevent or mitigate Dravet symptoms.

Gene therapy seems to be a promising treatment for Dravet syndrome and clinical trials with antisense oligonucleotides (ASOs)^8^ able to increase Na_v_1.1 expression are already ongoing. Specifically, for treatments like ASOs whose stability is limited^8^ or for those based on the continuous expression of transcriptional activator tools,^7,9^ it remains imperative to determine whether *Scn1a* is also required after the neurodevelopmental time-window or, alternatively, can be restricted to such a period to promote long-lasting beneficial effects on Dravet pathological symptoms throughout adulthood. To address this question, we employed a genetic strategy allowing to induce *Scn1a* gene haploinsufficiency at different postnatal time points (P2, P30 and P60) and ultimately to evaluate the functional relevance of physiological expression of functional Na_v_1.1 in the adult brain.

We found that early postnatal induction (P2) of *Scn1a* haploinsufficiency recapitulated phenotypes of classic Dravet syndrome models and that, similarly, P30- and P60-induced mice displayed seizures and SUDEP, accompanied with GABAergic interneurons dysfunction that was evident in all three groups of mice at their specific symptom onset. Behavioural alterations, including hyperactivity and anxiety and alterations in social and environment interaction were comparable in the three groups of mice. Interestingly, cognitive performance was better in P30- and P60-induced mice compared to early postnatal induced mice, with scores in the latest induction group comparable to the controls. These findings highlight the key role of Na_v_1.1 normal levels during adulthood providing fundamental knowledge for the design of efficacious therapeutic strategies.

## Materials and methods

### Study design

This study aimed to test the hypothesis that maintaining physiological levels of Na_v_1.1 until the adulthood in a conditional Dravet syndrome mouse model is sufficient to block or at least ameliorate the severity of the pathological symptoms of the syndrome. The experiments were designed to achieve a power > 0.8 with an α = 0.05. The 3Rs guidelines for animal welfare were followed. Outliers were not excluded and at least three independent repetitions were performed. Exclusion criteria were applied for the recordings (see later) and for animals in the behavioural tests (see later). All the experiments were randomized and mutant mice of each litter were randomly associated to the tamoxifen or vehicle treatments. Researchers were blinded during the experimental execution and the analysis, as the vials containing tamoxifen or vehicle were prepared by a person external to the project. Confounders were not controlled. Each animal was considered as an experimental unit in EEG, survival and behavioural test, while each cell patched was considered as an experimental unit for the recordings.

### Mice

Mice were maintained at San Raffaele Scientific Institute Institutional mouse facility (Milan, Italy) and supplied with autoclaved food and water. The conditional *Scn1a^floxedA^*^1783*V/+*^ mice [B6(Cg)-Scn1atm1.1Dsf/J, The Jackson Laboratory, strain no. 026133] were bred to UBC-Cre-Ert2 mice [B6.Cg-Ndor1Tg(UBC-cre/ERT2)1Ejb/1J The Jackson Laboratory, strain no. 007001]. Mice were genotyped with the following primers: *Scn1a^floxedA1783V/+^*: 24472_ Forward GCAACTCTTCACATGGTACTTTCA; 24473_Common GCACCTCTCCTCCTT AGAACA; 24489_Mutant Forward GGAGAAACACGAGCAGGAAG; UBC-CRE-ERT2: 25285_Transgene Forward GACGTCACCCGTTCTGTTG; oIMR7338_Internal Positive Control Forward CTAGGCCACAGAATTGAAAGATCT; oIMR7339_Internal Positive Control Reverse GTAGGTGGAAATTCTAGCATCATCC; oIMR9074_ Transgene Reverse AGGCAAATTTTGGTGTACGG. PCR protocols available on Jackson website were used. To assess the efficiency of Cre-mediated recombination, mice carrying the UBC-Cre-ERT2 allele were crossed with mice carrying the Ai9 mice (The Jackson Laboratory, strain no. 007909). All procedures were performed according to protocols approved by the internal IACUC and reported to the Italian Ministry of Health according to the European Communities Council Directive 2010/63/EU.

#### Generation of conditional *Scn1a^floxedA1783V/+^*;UBC-Cre-ERT2 knock-in mice

Breeding pairs consisted of heterozygous male *Scn1a^floxedA1783V/+^* and heterozygous female UBC-Cre-ERT2 mice. A1783V mutation was induced in *Scn1a^floxedA1783V/+^*;UBC-Cre-ERT2 mice at three different postnatal (P) time points, P2, P30 and P60 by tamoxifen administration, and compared to mice with no gene mutation induction. Tamoxifen (T5648-Sigma) was dissolved in corn oil (C8267-Sigma) at 42°C under vigorous shaking and injected intraperitoneally daily for 4–5 consecutive days (100 µg/day at P2; 75 mg/kg at P30 and P60). Corn oil was injected in vehicle control animals. Concomitantly, to investigate the efficiency of recombination, Ai9;UBC-Cre-ERT2 mice were intraperitoneally injected with tamoxifen following the same dose and treatment schedule used for the generation of conditional *Scn1a^floxedA1783V/+^*.

To assess the efficiency of A1783V mutation induction, RNA was extracted from cerebral cortices isolated from three *Scn1a^floxedA1783V/+^*;*UBC-Cre-E*RT*2* mice from each induction condition (P2, P30, P60 and no induction) using TRI reagent (Merck) according to the manufacturer’s instructions. After DNase I treatment to digest genomic DNA, RNA was converted to cDNA using the ImProm-II Reverse Transcription System (Promega). The cDNA was then amplified using the following primers: *Scn1a* A1783V_F GTTCCAAATCACCACCTCTGCG and *Scn1a* A1783V_R GGCGTCAGGGTCGAACTTCT, using GoTaq DNA polymerase (Promega). The 307 bp amplified products were gel-extracted and TA cloned in PCR2.1 vector (ThermoFisher). Twenty colonies from each condition were miniprepped and sequenced at Microsynth. SnapGene was used to align sequences to *Scn1a* coding sequence and localize A to V single nucleotide mutation.

### Immunostainining

Mice were anaesthetized and perfused with paraformaldehyde 4%/PBS. Brains were extracted and post-fixed overnight in paraformaldehyde 4%/PBS, then cryoprotected in sucrose 30%/PBS and frozen in isopentane (Sigma). Coronal brain sections (50-µm thick) were cut with a cryostat and free-floating sections were used for immunofluorescence analysis, as previously shown.^7,10^ Briefly, after a quick wash in PBS, brain sections were blocked in a solution containing 10% donkey serum, 0.3% Triton X-100 in PBS and then incubated overnight with anti-RFP primary antibody (rabbit, 1:1000, MBL; code: PM005) diluted in the same blocking solution. Following washing in PBS, sections were incubated with anti-rabbit-546 secondary antibody (1:1000, Thermofisher; code: A10040) and the nuclear marker Hoechst (1:1000, Thermofisher; code: H1399). Pictures were taken with Leica TCS SP8 confocal microscope, using a 40× magnification lens with a *z*-step size of 1 µm. ImageJ 1.53a cell counter tool was used for tdTomato+ and Hoechst+ cell counting. Three to five confocal images for each mouse, three mice per group.

*Post hoc* immunostaining of biocytin-filled neurons was performed by adapting a previously established protocol.^11^ Briefly, immediately after electrophysiological recordings brain slices were fixed for 1 h in paraformaldehyde 4%/PBS at 4°C, then washed in PBS and blocked in a solution containing 5% donkey serum, 0.3% Triton X-100 and 1% bovine serum albumin in PBS for 1 h at 4°C. Next, they were incubated overnight at 4°C with anti-parvalbumin (PV) primary antibody (rabbit, 1:500, Swant; code: PV27A) diluted in the same blocking solution. After washing in PBS, slices were incubated for 1 h at room temperature (T_R_) with the secondary antibody anti-rabbit-488 (1:500, Thermofisher; code: A21206), streptavidin-647 (1:500, Thermofisher; code: S21374) and Hoechst (1:1000, Thermofisher; code: H1399), then washed in PBS and mounted on a glass slide for visualization at the Leica TCS SP8 confocal microscope. Pictures were taken through the LAS X integrated software using 10× and 40× magnification lenses with a *z*-step size of 2–3 µm.

### Survival monitoring

Mice were monitored daily for survival up to P120 without knowing the genotype and treatment. Survival data were analysed for each genotype and survival curves were generated using Prism (GraphPad Software, Inc., CA, USA).

### Video-EEG recordings and analysis

Mice were surgically implanted under 1.5% isoflurane anaesthesia and stereotaxic guidance, with a wireless radiofrequency transmitter (ETA-F10, Data Science International, DSI) placed subcutaneously in the back. The recording electrodes were implanted bilaterally in the somatosensory cortex (from bregma, mm: anteroposterior, −1; lateral, ±1) and fixed with white glass ionomer cement. EEG signal was wireless transmitted to MX200 (DSI), digitally recorded using Ponemah (DSI), and sampled at a frequency of 500 Hz. For EEG analysis, Neuroscore software (DSI) was used. For spontaneous seizures detection, EEG traces were first band-pass filtered between 5 and 70 Hz. After artefact exclusion based on signal amplitude, seizures were defined as transient changes (5–700 ms) in EEG traces [>5 standard deviations (SD)], using an automated protocol. Video recordings were then visually inspected to confirm seizures. All mice were video-EEG recorded continuously (24/7) at least for 14 days; starting from around P50–P60 in the case of P2- and P30-induced. P60-induced mice were video-EEG recorded starting 1 week before tamoxifen treatment and monitored long term until 80 days post-tamoxifen treatment.

### Seizure thermal induction

We adopted a previously published protocol,^10^ with some modifications. Briefly, mice were placed in a glass beaker and heated with an infrared heat lamp (HL-1, Phisitemp) to gradually increase the body temperature, controlled by a TCAT-2DF thermo controller (Phisitemp). Mouse rectal temperature was continuously controlled by using a RET-3 probe (Phisitemp). Mice were recorded at baseline for 15 min, then seizures were evoked by increasing the body temperature by 0.5°C every 30 s. The heating lamp was then switched off to allow recovery and mice were monitored until temperature returned to the baseline.

### *Ex vivo* electrophysiology

For single cell electrophysiological recordings, hippocampal brain slices were obtained as previously described.^10^ Briefly, at each time point mice of the two experimental groups were euthanized following deep isoflurane anaesthesia and brains were isolated in ice-cold cutting solution [modified artificial CSF containing in mM: 92 sucrose, 87 NaCl, 2.5 KCl, 1.25 NaH_2_PO_4_, 25 NaHCO_3_, 25 glucose and 10 MgSO_4_, aerated with 95% O_2_ and 5% CO_2_ (pH 7.4)]. Coronal hippocampal sections (350-μm thick) were cut using a Leica VT 1200 vibratome. The slices were allowed to recover in the cutting solution at 32°C for 15 min and then at room temperature for 15 more minutes before being moved in a chamber filled with the recording solution [containing in mM: 125 NaCl, 25 NaHCO_3_, 10 D-glucose, 2.5 KCl, 1.25 NaH_2_PO_4_, 2 CaCl_2_ and 1 MgCl_2_, (pH 7.4)] maintained at room temperature for at least 30 min before recording. For electrophysiological recordings, hippocampal slices were placed in the recording chamber beneath a 40× water immersion lens and visualized using infrared differential interference contrast (IR-DIC) video microscopy on an Olympus upright microscope. Inhibitory interneurons of the CA1 region of the hippocampus were recorded, identified by the location of their somata in the stratum oriens (s.o.). *Bona fide* PV+ cells were found at the border between the stratum pyramidale (s.p.) and stratum oriens, recognized by their large soma compared to pyramidal excitatory neurons. Their identity was confirmed after recording, based on their typical firing properties: high firing frequency (>50 Hz at 350 pA) with little to no adaptation and no depolarization block (or, for induced *Scn1a* haploinsufficiency group, appearing after 400 pA stimulation step), fast afterhyperpolarization (AHP) and decay slope, high AHP amplitude, and they were classified as fast spiking (FS) interneurons. All other interneurons recorded in the stratum oriens were grouped together and classified as non-fast spiking (non-FS) interneurons in contraposition to FS interneurons. FS interneuron identity of some cells patched was further confirmed by *post hoc* immunostaining against PV of cells filled during recording with 0.2% biocytin diluted in the intracellular solution. Whole-cell patch-clamp recordings were performed in continuous superfusion with carbogenated artificial CSF, maintained at a controlled temperature of 30–32°C. For current clamp (CC) recordings patch electrodes (2.5–3 MΩ) contained (in mM): 124 KH_2_PO_4_, 5 KCl, 2 MgCl_2_, 10 NaCl, 10 HEPES, 0.5 EGTA, 2 Na-ATP and 0.2 Mg-GTP (pH 7.25). Resting membrane potential (V_m_) was recorded for at least 2 min holding the potential at −70 mV for following measurements. To determine both the passive membrane properties and active firing properties, such as the action potential (AP) firing threshold and frequency, a current step protocol was used. The input-output (IO) curves were obtained by injecting 500 ms current steps of increasing amplitude from −50 to 800 pA (Δ = 10 pA) for FS neurons and from −50 to 600 pA for non-FS neurons. An AP was defined by a rising slope > 15 mV/ms and a peak exceeding −10 mV. Neurons with unstable or depolarized (> −50 mV) V_m_ and/or with a holding current > 200 pA when held at −70 mV were discarded. Bridge balance compensation was applied. All recordings were acquired using a Multiclamp 700B Amplifier (Molecular Devices), low-pass filtered at 10 kHz and digitized at 50–100 kHz using a Digidata 1550 D/A converter (Molecular Devices) controlled by the pCLAMP11 software (Molecular Devices). Access resistance (R_a_) was monitored continuously during the recording and cells with a R_a_ > 25 MΩ, or with a variation >20% were excluded from analysis.

Recordings were analysed using Clampfit 11.2 (Molecular Devices). Passive membrane properties were derived from the voltage response to the hyperpolarizing current steps of the current step protocol (average of five steps), as previously described.^7,10^

The voltage threshold for firing was determined through the phase-plane plot [plot of the time derivative of voltage (dV/dt) versus voltage] of the first AP elicited by the current step protocol.^12^ The threshold was defined as the first voltage value at which dV/dt exceeded 10 mV/ms. Rheobase was determined as the amplitude of the first current step at which an AP was fired, and the current threshold density was calculated by dividing the rheobase by the cell capacitance. The AP and AHP amplitude were obtained from the AP and AHP peak, respectively, relative to the voltage threshold. AHP delay was determined as the latency between the AP peak and AHP peak. The AP maximal rise and decay slopes were defined as the maximal and minimal values, respectively, of the time derivative of the voltage. The inflection rate was calculated as the slope of the phase-plane plot at AP threshold.^13^

### Behavioural tests

Behavioural tests were performed by the personnel of the Mouse Behavior Core Facility at San Raffaele Scientific Institute. Different cohorts of mice were tested in a total of eight rounds of behaviour, in which control mice were always tested. All the mice performed all behavioural tasks in the sequence and time indicated in the ‘Results’ section. Operators were blinded to the genotype during both the testing and the analysis. Mice experiencing tonic-clonic seizures during a test were excluded from the analysis of that specific test.

#### Marble burying test

Twelve glass marbles were placed on the bedding surface of a rat cage filled with 5 cm of fresh litter cage in three equidistant rows of four marbles each. Each mouse was placed into the centre of the cage with marbles and after 20 min they were returned to their home cages without moving or dislodging the marbles. The number of buried marbles was counted. A marble was considered buried when at least 2/3 of its surface was covered by litter. Litter was changed and marbles washed after each use.

#### Sociability

Mice were tested, using the three-chamber sociability test, in a 60 × 40 cm plexiglass box divided into three evenly split chambers by transparent walls with closable gates allowing free access to each chamber. The test consists of three consecutive 10-min phases.

##### Habituation phase

Test mice could freely explore the three empty chambers for 10 min through the open gates.

##### Phase 1: sociability

An unfamiliar mouse (Stranger 1) was placed inside a cylinder in one of the side chambers while an unfamiliar object (wire metal cylinder, diameter 10.5 cm, height 11 cm) was placed in the other side chamber, with the gates open to allow the test mouse to explore all three chambers for 10 min.

##### Phase 2: social novelty

A second unfamiliar mouse (Stranger 2) was placed inside the empty cylinder from Phase 1. The other chamber side remained as in Phase 1, with Stranger 1 now considered as familiar. The gates were open, allowing the test mouse to freely explore all three chambers for 10 min. Stranger mice were the same strain, sex and age-matched with test mice and were habituated to the wire cylinders in advance. To evaluate social behaviour and social memory, time spent in each chamber and time spent sniffing was scored. Trials were video-recorded using the video tracking software (Ethovision XT 17, Noldus).

#### Open field and novelty test

The test was conducted in a 50 × 50 open arena. Mice were allowed to freely explore the arena for 30 min. Distance moved (cm), mean speed (cm/s), time spent in the home, transition and exploration areas were evaluated. Subsequently, to test free exploratory behaviour, a novel object, a 50-ml Falcon tube was glued vertically in its centre and mice were observed for an extra 30 min. Distance moved (cm), mean speed (cm/s), time spent in the home, transition and exploration areas and time sniffing the novel object were evaluated. Trials were video-recorded and automatically analysed using the video-tracking software (Ethovision XT 17, Noldus).

Stereotypies and repetitive behaviours were analysed during the first 10 min of the open field test. Circling behaviour was scored automatically by the video-tracking software (Ethovision XT 17, Noldus), considering a complete 360° turn of nose angle with respect to the body centre as one event. The amount of time spent grooming and the total number of grooming events were measured manually. Grooming was defined as face-wiping, scratching and rubbing of head and ears, paw and fur licking. The number of jumps was manually counted by the operator.

#### Rotarod test

The rotarod test has been developed to measure motor coordination in mice and rats.^14^ Mice were assessed on both constant and accelerating speed horizontal rotarod (diameter ∼3 cm) for their balance activity. The latency to fall off the rod was the dependent variable analysed. The test was performed over five consecutive days. Each day, a training phase preceded the testing phase. The training phase consisted of three consecutive trials of 1 min each with intervals of 10 min. In the first trial, the rod was kept stationary while in the second and third trials, it was held at 5 rpm. Thirty minutes after the third training trial, mice were subjected to the test phase, consisting of four trials per day with intervals of 15 min, with rotation speed accelerated from 5 to 40 rpm in 300 s.

#### Morris water maze

Mice were trained in a circular pool of (Ø150 cm; 50 cm height). In the hidden platform (14 × 14 cm) version of the water maze, mice had to locate the platform in a fixed position. The test included an acquisition phase (18 trials, six per day, intertrial time 30–40 min) followed by a reversal phase during which the platform was moved to the opposite position (12 trials, six per day). Each trial lasts until the animal climbs onto the platform and stays there, or until 120 s have elapsed. To assess acquisition and platform reversal learning, the escape latency and the swim speed were scored. An automated software algorithm was used to classify video-tracked trials according to the predominant swimming strategy. Eight exclusive categories were defined to capture the improving spatial precision and efficiency during the learning process: circling, floating, wall-hugging, random swimming, scanning, chaining, focal searching and direct swims.^15^ Each mouse was categorized according to the most frequently shown strategy. The percentage of mice falling into each category was then computed for each experimental group of mice.

### Statistical analysis

Statistical analysis performed is shown in each figure legend. Deviation from normal distributions was assessed using D’Agostino-Pearson’s test, and the *F*-test was used to compare variances between two sample groups. Student’s two-tailed *t*-test (parametric) or the Mann-Whitney U-test (non-parametric) were used, as appropriate, to compare means and medians. Fisher’s exact test was used to analyse the contingency table. To compare two groups at different time points we used one-way ANOVA or two-way repeated measures ANOVA, followed by *post hoc* tests. Statistical analysis was carried out using Prism 8.0 (GraphPad Software, Inc., CA, USA). For electrophysiological parameters, statistical analysis was performed using SPSS Statistics 26 (IBM) and fitting data to a linear mixed-effect model (LMM) via the reduced maximal likelihood (REML) method to account for the random effect of mice in the evaluation of differences between experimental groups. Sidak’s *post hoc* test was used for means comparison and *P-*values are reported both in figure legends and Supplementary Tables 1 and 2. Statistical significance was reached when *P* < 0.05. Box plots display the median (internal horizontal line), first and third quartiles (upper and lower box edges) and minimal and max values (whiskers) of the data distribution. Circles represent individual data-points from each cell or mouse.

## Results

### Generation of delayed *Scn1a* gene inactivated mouse models

**Figure 1:**
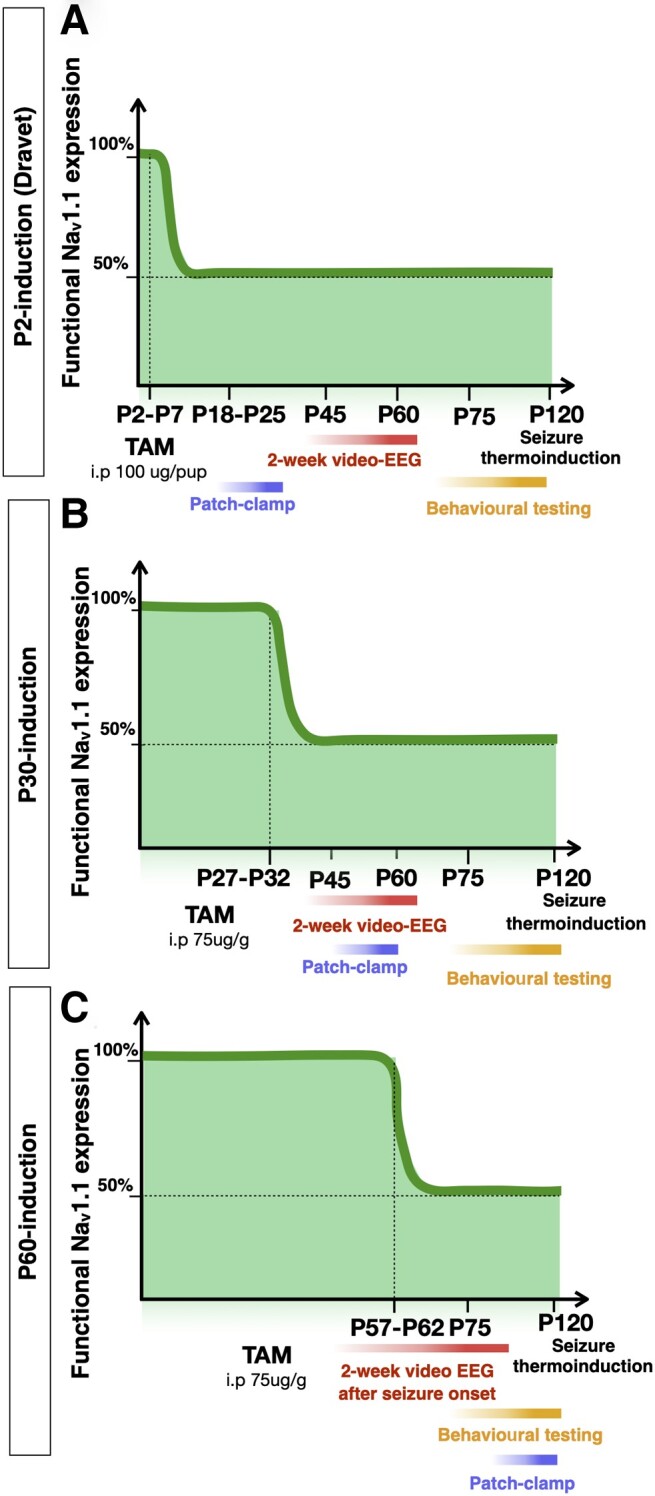
**Experimental design for *Scn1a* gene haploinsufficiency induction at different developmental time points.** *Scn1a^floxedA1783V/+^* mice were crossed with UBC-CreERT2^+/−^ mice and intraperitoneally injected with tamoxifen (TAM) at different postnatal days: P2 (**A**), P27 (**B**) and P57 (**C**) for five consecutive days. A cohort of mice from each experimental group was evaluated for spontaneous seizures: P2- and P30-induced mice were implanted with EEG transmitters at P45 and video-EEG recorded for two consecutive weeks. P60-induced mice were implanted at P45 before tamoxifen injection and video-EEG recorded for two consecutive weeks after seizure onset. Another cohort of male mice from each group was subjected to behavioural testing. After behavioural tests, mice were monitored for survival until P120 and then subjected to seizure thermal induction. A third cohort of mice from each group was euthanized for patch-clamp experiments at different time points: P18–P25 for P2-induced mice, P45–P60 for P30-induced, and P75–P120 for P60-induced.

To evaluate the importance of continued physiological expression of functional Na_v_1.1 after early postnatal development, we employed a conditional *Scn1a^floxedA1783V/+^* mouse model^16^ that enabled us to induce *Scn1a* gene haploinsufficiency at any desired time point. In this model, a C to T change at nucleotide 5348 in exon 26 can be conditionally inserted causing an alteration of the corresponding amino acid from alanine (A) to valine (V) at position 1783. Induction of this mutation since early stages of embryonic development by crossing the *Scn1a^floxedA1783V/+^* mice with CMV-Cre mice results in classic Dravet syndrome phenotype.^16^ To achieve temporal control on the postnatal induction of *Scn1a* gene haploinsufficiency, we crossed *Scn1^floxedA1783V/+^* mice with tamoxifen inducible Cre line (Cre-ERT2) expressed under the Ubiquitin promoter (UBC-Cre-ERT2^+/−^)^17^ obtaining *Scn1a^floxedA1783V/+^*;UBC-Cre-ERT2^+/−^ mice and relative controls (*Scn1a^floxedA1783V/+^*;UBC-Cre-ERT2^−/−^, *Scn1a^+/+^*;UBC-Cre-ERT2^−/−^ and *Scn1a^+/+^*;UBC-Cre-ERT2^+/−^, from here, referred to as control or Ctrl mice). By repeated tamoxifen injections (Supplementary Fig. 1A), we induced heterozygous expression of *Scn1a* mutant allele at different postnatal time points, in juvenile mice at P30 and adult mice at P60 and compared their phenotypes to those with perinatal (P2) gene inactivation, that should resemble Dravet mice (Fig. 1A–C). Induction of A1783V mutation in Na_v_1.1 does not alter its expression.^16^ Therefore, to ascertain if the tamoxifen administration efficiently induced *Scn1a* mutant allele expression in a sufficient number of cells, the same induction treatment was delivered in Ai9;UBC-Cre-ERT2^+/−^ mice at the same postnatal ages (P2, P30 and P60). Immunohistochemistry analysis confirmed tamoxifen-induced Cre recombination occurred efficiently throughout different brain areas, including the cerebral cortex, the hippocampus and striatum (Supplementary Fig. 1B). To further confirm A1783V mutation induction, RNA from cerebral cortex of three mice of each experimental group was extracted and processed for cDNA amplification and sequencing (Supplementary Fig. 1C).^18^ We were able to detect A1783V mutant mRNA molecules at all the induction points with expected proportion (between 40% and 50% of total *Scn1a* mRNA) (Supplementary Fig. 1C). No *Scn1a* mutant mRNA molecule was detected in cortices from the control group (Supplementary Fig. 1C).

### Delayed induction of Na_v_1.1 haploinsufficiency elicits epileptic phenotype comparable to Dravet mice

**Figure 2:**
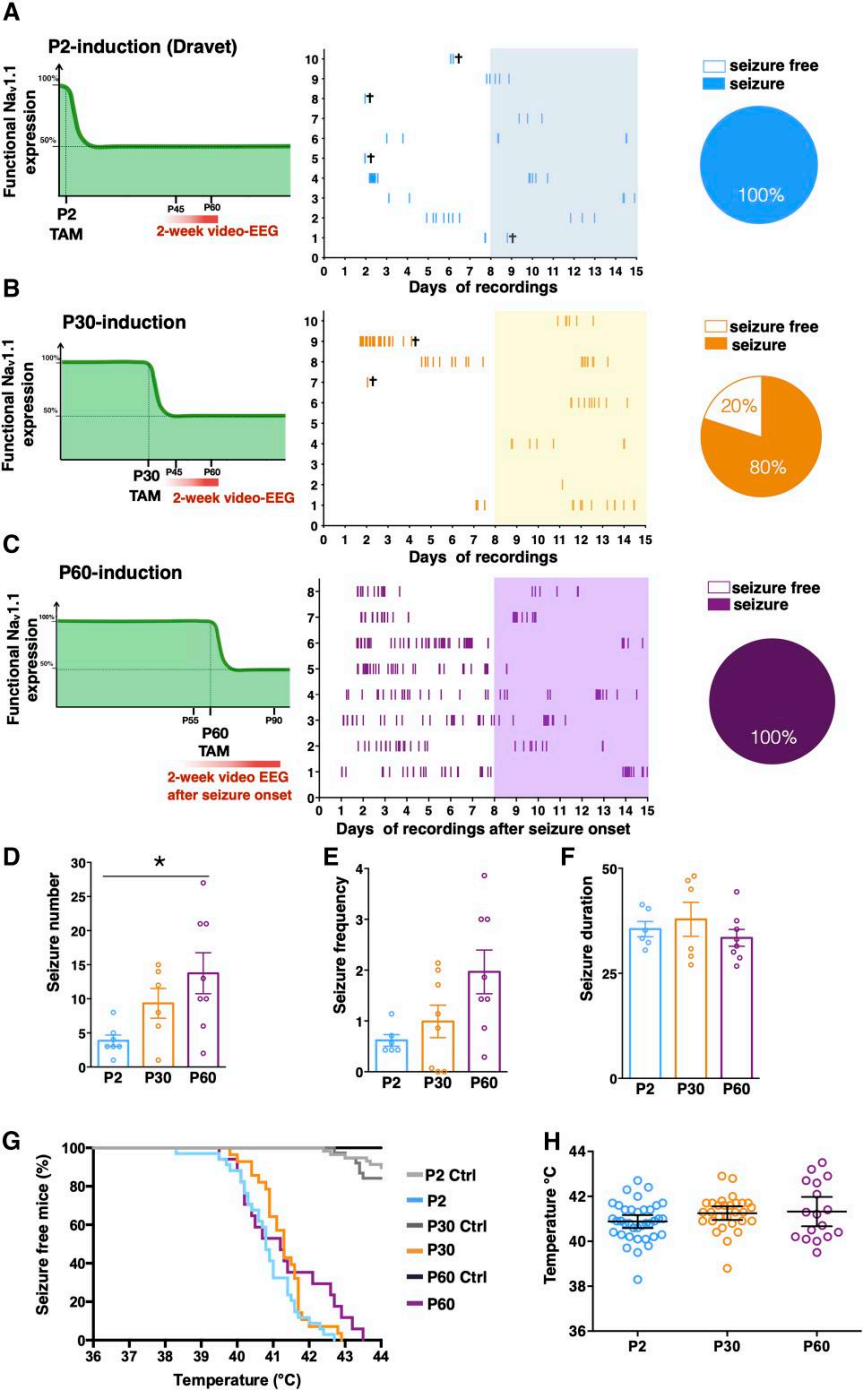
**Delayed induction of Na_v_1.1 haploinsufficiency elicits epileptic phenotype comparable to Dravet mice.** Schematics depicting the experimental design for P2 (**A**, *left*), P30 (**B**, *left*) and P60 (**C**, *left*) inactivation of *Scn1a* allele. Raster plot showing all generalized tonic-clonic seizures (Racine scale stages 4 and 5) in *Scn1a^floxedA1783V/+^* × UBC-CreERT2^+/−^ mice injected with tamoxifen (TAM) at P2 (**A**, *middle*; *n* = 10), P30 (**B**, *middle*; *n* = 10) and P60 (**C**, *middle*; *n* = 8) and subjected to 2 weeks of video-EEG recordings (mouse death for SUDEP during recordings is indicated). Pie charts showing the proportion of P2- (**A**, *right*), P30- (**B**, *right*) and P60- (**C**, *right*) *Scn1a* inactivated mice with or without spontaneous seizures. Cumulative seizure number (**D**), average daily seizure frequency (**E**) and average seizure duration (**F**) in the second week of video-EEG recordings (indicated with coloured boxes in the raster plot) in P2-, P30- and P60-*Scn1a* induced mice. Data are shown as mean ± standard error of the mean (SEM), with circles representing individual mice. *P = 0.016, one way-ANOVA followed by Tukey’s. (**G**) Percentage of P2-, P30- and P60-*Scn1a* induced mice and relative control mice remaining seizure-free after thermal induction (P2 Ctrl, *n* = 60; P2, *n* = 37; P30 Ctrl, *n* = 38; P30, *n* = 29; P60 Ctrl, *n* = 13; P60, *n* = 17; ****P < 0.0001; Mantel-Cox log-rank test). (**H**) Histogram showing the comparison of the average temperature at which the seizure occurred. Data are shown as mean ± SEM, with circles representing individual mice. Ctrl = control; SUDEP = sudden unexpected death in epilepsy.

We proceeded to assess the effect of delayed induction of Na_v_1.1 haploinsufficiency on the development of spontaneous seizures, a prominent feature of Dravet mice. EEG signals from somatosensory cortex coupled with video monitoring (video-EEG analysis) were recorded continuously for at least 2 weeks. P2- and P30-induced mice were implanted with wireless transmitters when they reached a suitable weight (∼20 g, around P40–P45). Conversely, *Scn1a^floxedA1783V/+^*;UBC-Cre-ERT2^+/−^ mice intended to be induced at P60 could be implanted with EEG transmitters before mutation expression (around P50–P55) and thus, we could detect seizure onset and record EEG activity at least up to 2 weeks after that. Like classic Dravet mice, all P2-induced mice (10 out of 10) developed tonic-clonic spontaneous seizures in the 2 weeks of video-EEG recordings and four mice died of SUDEP (Fig. 2A). Similarly, 80% of P30-induced mice developed spontaneous seizures with parameters comparable to those of the P2-induced group of mice and two died of SUDEP (Fig. 2B). In P60-induced mice, seizures started between 10–30 days after tamoxifen administration (Supplementary Fig. 2A). Mice experienced seizures at high frequency in the first week after the onset, these became sparser in the second week and acquired a rhythmic frequency in the chronic phase (Fig. 2C and Supplementary Fig. 2B). Quantification of seizure number and frequency in the second week of recording of three groups of mice show no significant difference between P2 and P30 (Fig. 2D and E), while a significant increase in the number and a trend in the frequency was observed between P2 and P60, likely due to the different phases of the pathology that were observed. No difference between seizure duration was observed in the three groups of mice (Fig. 2F). Age-matched control mice were recorded for 2 weeks but spontaneous seizures were never detected (data not shown).

Mice of the three groups and their relative controls were subjected to hyperthermia to induce seizures. While only few control mice showed hyperthermia-induced seizure and only at a very high temperature of induction (Fig. 2G), all P2-, P30- and P60-induced mice experienced a tonic-clonic seizure when body temperature was raised up to 44°C (Fig. 2G), with no significant difference in the temperature of induction among the three groups (Fig. 2H).

**Figure 3:**
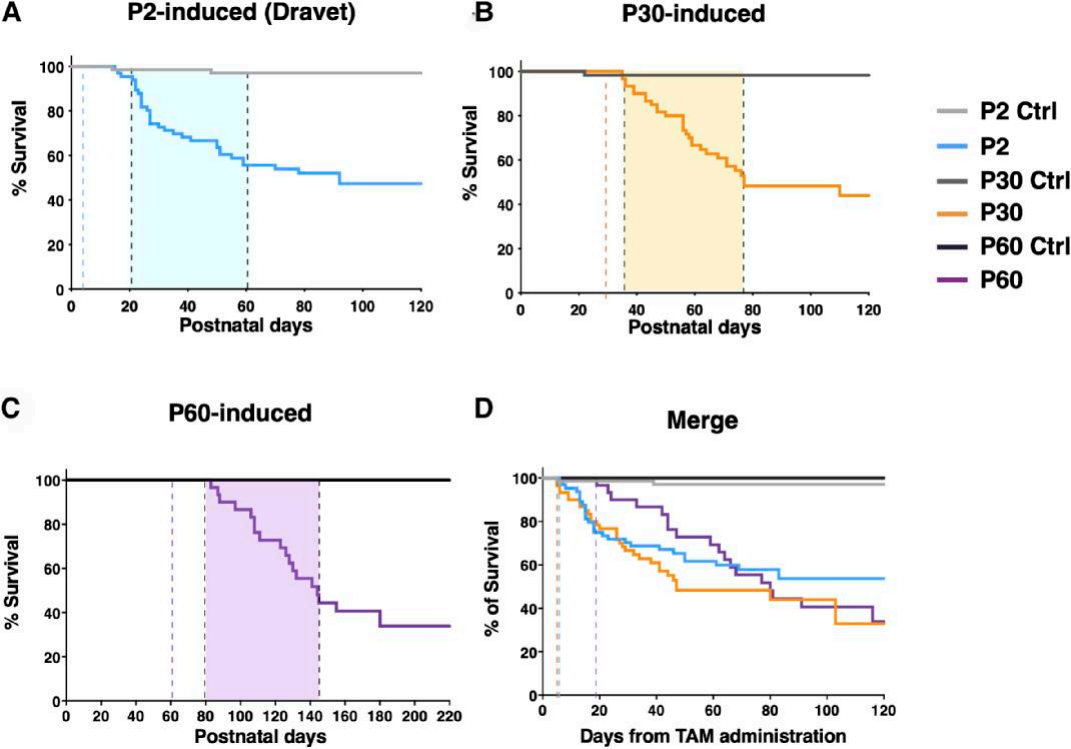
**Maintenance of physiological expression of Na_v_1.1 until P30 or P60 does not protect from SUDEP.** (**A**) Survival curve of P2-Ctrl (Control) (*n* = 69) and P2 (*n* = 66) *Scn1a* inactivated mice. ****P < 0.0001, Log-rank Mantel-Cox test. (**B**) Survival curve of P30-Ctrl (*n* = 61) and P30 (*n* = 60) *Scn1a* inactivated mice. ****P < 0.0001, Log-rank Mantel-Cox test. (**C**) Survival curve of P60-Ctrl (*n* = 18) and P60 (*n* = 30) *Scn1a* inactivated mice. ***P = 0.001, Log-rank Mantel-Cox test. The first dotted line indicates the start of tamoxifen (TAM) treatment while the coloured box starts at the onset of seizures. (**D**) Survival curve of P2-Ctrl (*n* = 69), P2 (*n* = 66), P30-Ctrl (*n* = 61), P30 (*n* = 60), P60-Ctrl (*n* = 18) and P60 (*n* = 30) mice, considering the elapsed days from the end of tamoxifen treatment. The dotted lines correspond with the onset of seizures. *P* = 0.29, Log-rank Mantel-Cox test. SUDEP = sudden unexpected death in epilepsy.

Dravet mice die of SUDEP, with higher incidence in the onset period. Accordingly, in P2-induced mice, mortality started around the second week of age and peaked around the third (Fig. 3A). P30-induced mice started to die at around P40 and P60-induced mice at around P80 (Fig. 3B and C). When we normalized the curves on the time since tamoxifen administration, we did see an overlapping of the mortality curves with a non-significant trend toward a delay in the first events for P60-induced mice (Fig. 3D).

Taken together, our data show that delayed induction of *Scn1a* gene haploinsufficiency at P30 and even at P60 determines the appearance of both spontaneous and hyperthermia-induced seizures as well as SUDEP phenotype in mice.

### Interneuron GABAergic hypoexcitability underlies symptom onset in lately induced *Scn1a* haploinsufficient mice

**Figure 4:**
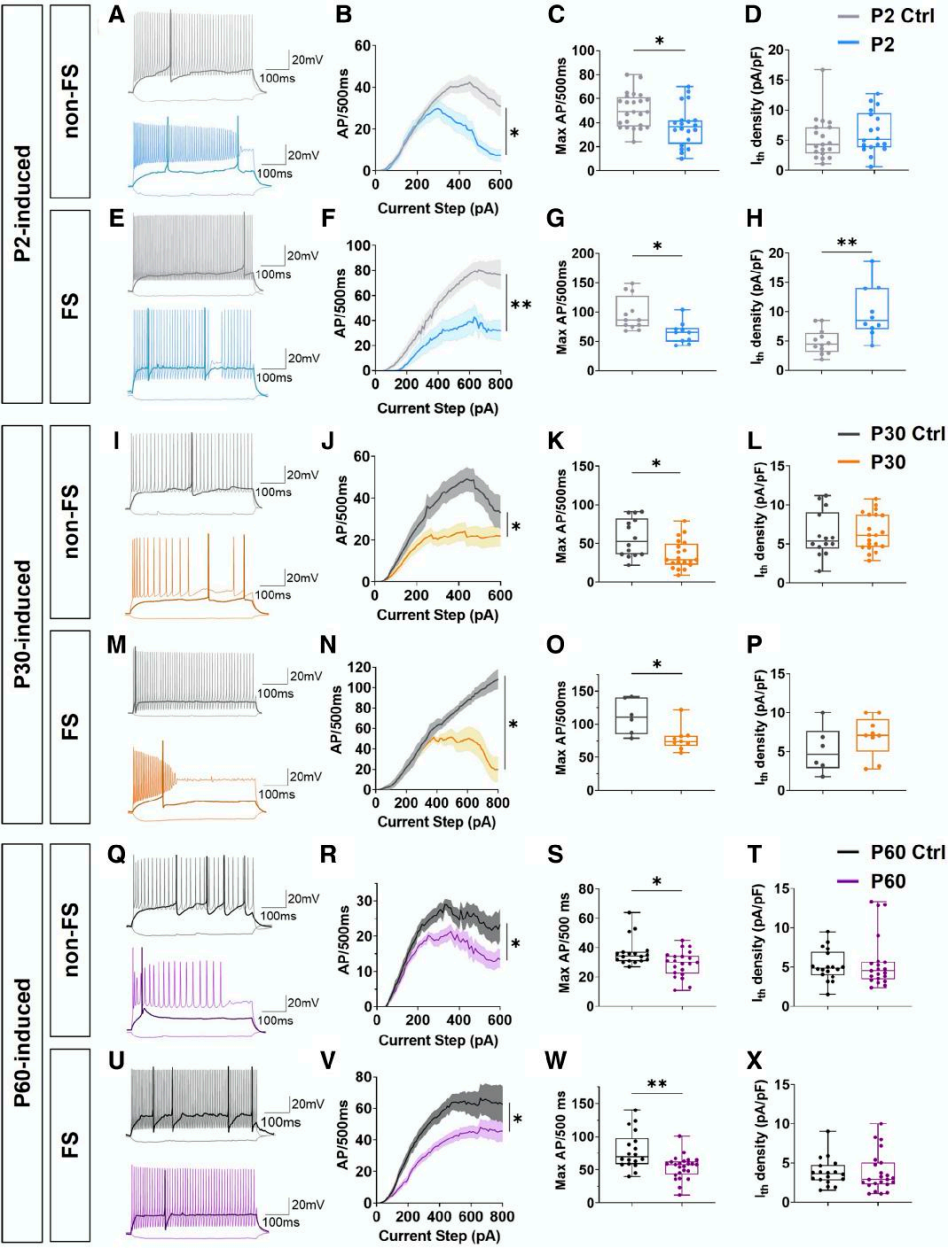
**GABAergic interneuron dysfunction underlies symptom onset in delayed *Scn1a* haploinsufficiency induced mice.** (**A**) Representative traces of non-fast spiking (non-FS) and (**E**) FS interneurons in CA1 of P2 Ctrl (Control) and P2-induced mice recorded at P18–P25. The voltage responses to one hyperpolarizing current step (−50 pA) and two depolarizing steps (rheobase, thicker trace, and +350 pA for non-FS or +500 pA for FS interneurons) are shown. (**B** and **F**) Input-output (IO) curve of the average firing rates in response to increasing current steps for non-FS and FS interneurons of the two experimental groups (non-FS *P = 0.028, LMM via REML, *n* = 19c/4a P2 Ctrl and 18c/4a P2-induced; FS ** *P* = 0.004, LMM via REML, *n* = 12c/5a P2 Ctrl and 1°c/4a P2-induced). (**C** and **G**) Maximal firing rate extrapolated from IO curve (non-FS *P = 0.017 and FS *P = 0.047, LMM via REML). (**D** and **H**) Current threshold density (FS **P = 0.002, LMM via REML). (**I** and **M**) Representative traces of non-FS and FS interneurons of P30 Ctrl and P30-induced mice recorded at P45–P60. The voltage responses to one hyperpolarizing current step (−50 pA) and two depolarizing steps (rheobase, thicker trace, and +350 pA for non-FS or +500 pA for FS interneurons) are shown. (**J** and **N**) IO curve of the average firing rates in response to increasing current steps for non-FS and FS interneurons in CA1 (non-FS *P = 0.044 and FS *P = 0.029, LMM via REML). (**K** and **O**) Maximal firing rate (non-FS *P = 0.047 and FS *P = 0.012, LMM via REML). (**L** and **P**) Current threshold density. (**Q** and **U**) Representative traces of non-FS and FS interneurons in CA1 of P60 Ctrl and P60-induced mice recorded at P85–P105. The voltage responses to one hyperpolarizing current step (−50 pA) and two depolarizing steps (rheobase, thicker trace, and +350 pA for non-FS or +500 pA for FS interneurons) are shown. (**R** and **V**) IO curve of the average firing rates in response to increasing current steps for non-FS and FS interneurons (non-FS *P = 0.023 and FS *P = 0.015, LMM via REML). (**S** and **W**) Maximal firing rate (non-FS *P = 0.020 and FS ***P < 0.001, LMM via REML). (**T** and **X**) Current threshold density. In IO curves, data are presented as mean ± standard error of the mean (SEM) while box plots display the median (internal horizontal line), first and third quartiles (upper and lower box edges) and minimal and max values (whiskers) of the data distribution. Circles represent individual data-points from each cell. AP = action potential; LMM = linear mixed-effect model; REML = reduced maximal likelihood.

Impairment in excitability of different subtypes of GABAergic interneurons due to reduction of Na+ currents is a hallmark of heterozygous *Scn1a* knock-out Dravet mice,^19-23^ with a more prominent defect in PV+ FS interneurons.^19,20^ A1783V mutation has been reported to alter both activation and inactivation of the Na_v_1.1 channel, mostly preserving the sodium current peak density.^24^ However, the characteristic firing defect of interneurons was observed when the mutation was induced in brain slices.^24^ To understand if interneuron dysfunction also characterizes symptom onset in mice with late induction of Na_v_1.1 carrying A1783V mutation, we performed whole-cell patch clamp recordings in CA1 area on acute brain slices from P2-, P30- and P60-induced mice and relative controls. The occurrence of first SUDEP events was considered as a sign of symptom onset for each of the three experimental groups: P18–P25 for P2-induced, P40–P45 for P30 and P85–105 for P60, respectively. GABAergic interneurons patched in CA1, were classified as fast spiking (FS) or non-FS based on their firing pattern, and analysed separately. Interneuron identity was also confirmed in a fraction of cells by *post hoc* immunostaining against PV after neuronal filling with biocytin (Supplementary Fig. 3). A reduced number of evoked APs, particularly at high stimulation intensities, was observed in both FS and non-FS interneurons in P2-, P30- and P60-induced mice compared to age-matched controls (Fig. 4A, B, E, F, I, J, M, N, Q, R, U and V), confirming that interneurons are hypoexcitable at symptom onset. Accordingly, in both non-FS and FS interneurons, maximal number of APs was reduced (Fig. 4C, G, K, O, S and W) in *Scn1a* haploinsufficiency induced mice with respect to their controls at each induction time point. Moreover, in FS interneurons at P2 induction time point an increase in current threshold was also detected (Fig. 4H), a tendency that was also observed at P30 induction time point (Fig. 4P), compared to controls. Other active and passive cell properties are reported (Supplementary Figs 4 and 5 and Supplementary Tables 1 and 2), showing a trend in FS interneurons toward a slower AP rise and decay time in *Scn1a* haploinsufficiency induced mice, which could reflect the defect in channel activation and inactivation phases.^24^ Taken together, our data suggest that GABAergic interneuron dysfunction underlies Dravet symptom onset even in late induced *Scn1a* haploinsufficient mice.

### Delayed *Scn1a* inactivation provokes behavioural alterations with mild or no effect on cognitive performance

Together with seizures, Dravet patients display disabling behavioural alterations, including hyperactivity, anxiety, motor delay, social interaction and cognitive impairment.^25,26^ To test the effect of delayed induction of *Scn1a* gene haploinsufficiency on those specific behavioural alterations, we subjected P2-, P30- and P60-induced male mice and a mixed group of control male mice, between 2.5 and 3.5 months of age, to a battery of behavioural tests (Supplementary Fig. 6). In the rotarod test, P2-induced mice did not show relevant alterations in motor coordination and balance in comparison to control mice in both constant velocity and acceleration (Supplementary Fig. 7), thus P30- and P60-induced mice were not tested for motor alterations.

**Figure 5:**
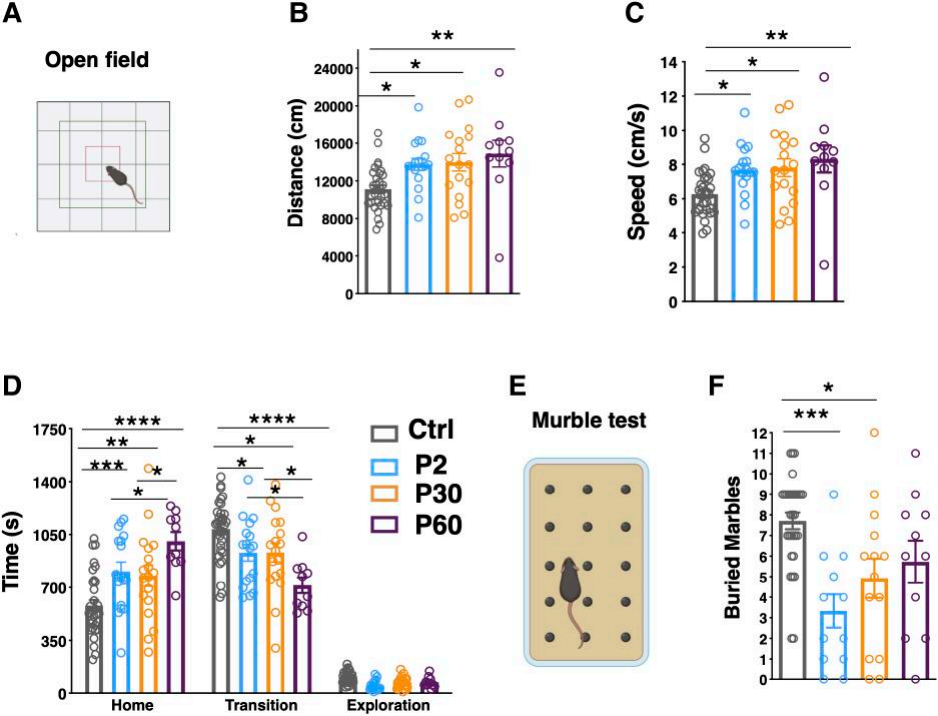
**Delayed *Scn1a* haploinsufficiency induction provokes hyperactivity and anxiety.** (**A**) Scheme of the open field arena created with Biorender.com. (**B**) Distance travelled, (**C**) velocity and (**D**) time spent in home, transition and exploration areas of the arena. Control (Ctrl) *n* = 32, P2 *n* = 17, P30 *n* = 18, P60 *n* = 11; ****P < 0.0001, ***P < 0.0005 **P < 0.005, *P < 0.05; two-way ANOVA followed by Tukey’s multiple comparison test. (**E**) Scheme of the Marble test created with Biorender.com. (**F**) Number of buried marbles. Ctrl *n* = 31, P2 *n* = 12, P30 *n* = 14, P60 *n* = 11; ***P = 0.0004, *P = 0.0274, Kruskall-Wallis test followed by Dunn’s multiple comparison test. Data are shown as mean ± standard error of the mean (SEM), with circles representing individual mice.

To investigate hyperactivity and anxiety behaviour, the four experimental mouse groups were observed in the open-field test (Fig. 5A). P2-induced mice displayed hyperactive behaviour in the arena with mice travelling significantly farther and faster than control mice (Fig. 5B and C), and P30- and P60-induced mice displayed the same tendency (Fig. 5B and C). In the arena, anxious mice usually spend more time in the periphery (home) compared to the other regions in which the arena was divided (transition, exploration) (Fig. 5A). P2- and P30-induced mice appeared more anxious compared to control mice as they spent more time in the home compared to the control group (Fig. 5D) and P60 mice spent even more time in the home compared to the other groups (Fig. 5D). No difference in repetitive behaviours, including body rotations and grooming, occurring during the open field, was reported (Supplementary Fig. 8A and B). To test exploratory behaviour, a novel object was placed in the centre of the arena and no difference in the behaviour of P2-induced versus control mice was detected (Supplementary Fig. 8C–G). Increased anxiety in P2- and P30-induced mice compared to controls was also confirmed in the marble burying test, where P60-induced mice showed a non-significant trend toward a better performance in comparison to P2-, burying a similar number of marbles to the control group (Fig. 5E and F).

**Figure 6:**
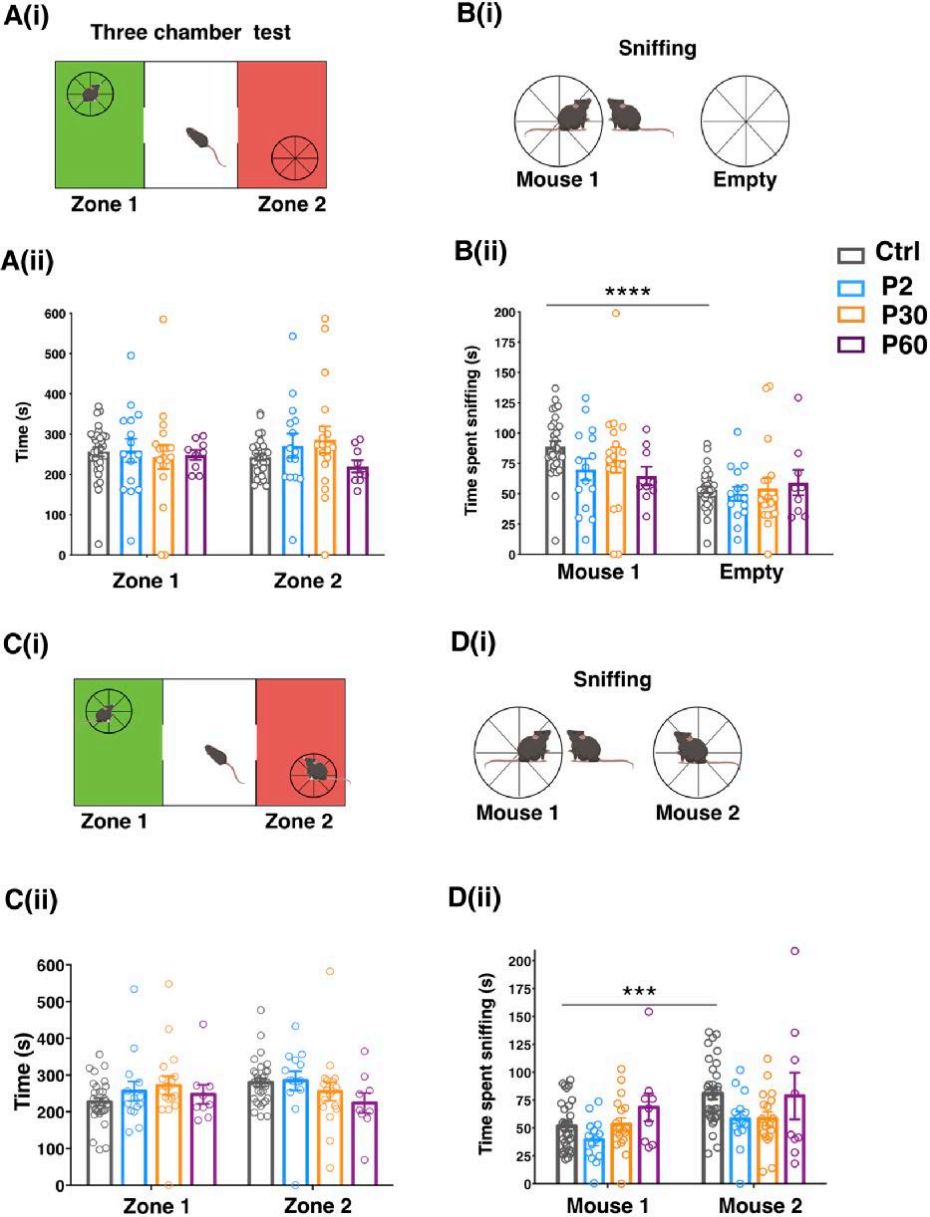
**Delayed *Scn1a* haploinsufficiency induction alters social interactions.** [**A**(**i**)] Scheme of the three-chamber test for social behaviour evaluation created with Biorender.com. [**A**(**ii**)] Time spent in Zone 1 and Zone 2 chambers when an unfamiliar mouse is placed in the Zone 1 chamber. [**B**(**i**)] Scheme of sniffing during social behaviour evaluation created with Biorender.com. [**B**(**ii**)] Time spent in sniffing. ****P < 0.0001; two-way ANOVA followed by Sidak’s multiple comparison test. [**C**(**i**)] Scheme of the three-chamber test for social memory evaluation created with Biorender.com. [**C**(**ii**)] Time spent in the Zone 1 and Zone 2 chambers when the familiar mouse is in Zone 1 and an unfamiliar mouse is placed in Zone 2. [**D**(**i**)] Scheme of sniffing during social memory evaluation created with Biorender.com. [**D**(**ii**)] Time spent in sniffing. ***P < 0.0005, two-way ANOVA followed by Sidak’s multiple comparison test. Data are shown as mean ± standard error of the mean (SEM) with circles representing individual mice. Control (Ctrl) *n* = 32, P2 *n* = 15, P30 *n* = 18, P60 *n* = 9.

To test social ability, we performed the three-chamber test [Fig. 6A(i)]. In the habituation trial, no preference for any of the areas of the arena was shown for the four groups of mice (Supplementary Fig. 9A and B) and when an unmet mouse was added to Zone 1, no preference was observed in terms of total time in each area [Fig. 6A(ii)]. However, when we measured the exact time spent sniffing the novel mouse and empty cage, control mice displayed a clear preference for the mouse versus the empty cage, while P2-, P30- and P60-induced mice did not [Fig. 6B(i and ii)], showing the sociability defect characteristic of Dravet mice.^10,26^ When a stranger age- and sex-matched mouse was added to Zone 2, again no preference was observed in terms of total time spent in Zone 1/Zone 2 [Fig. 6C(i and ii)] but, as expected, control mice preferred sniffing the second mouse in comparison to the first one, while delayed induced mice did not [Fig. 6D(i and ii)], highlighting no propension for social novelty. Our results suggest that the social interaction deficit characteristic of Dravet mice is still evident in mice with delayed induction of *Scn1a* haploinsufficiency at P30 and P60. In addition, P2-induced mice present a social novelty defect,^26^ that was also evident upon delayed induction of the *Scn1a* gene mutation.

**Figure 7:**
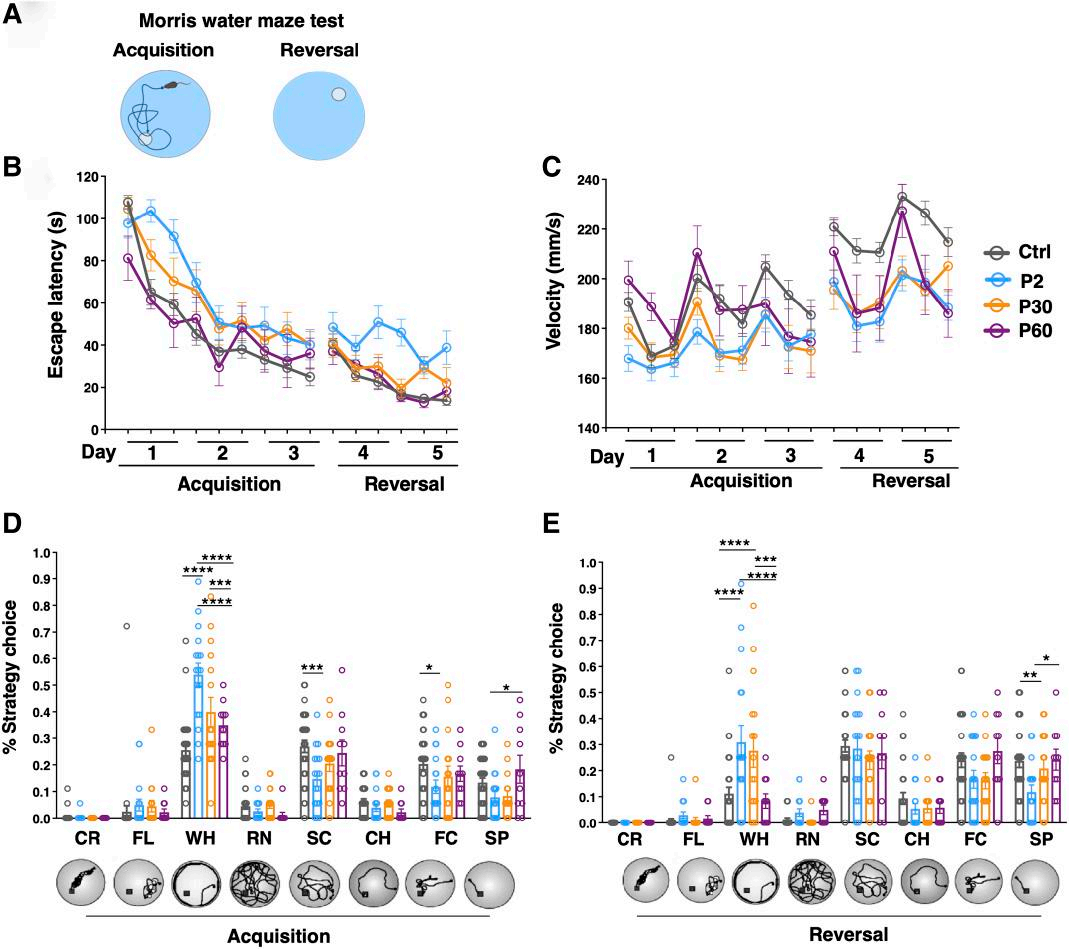
**Delayed *Scn1a* haploinsufficiency induction has mild or no effect on cognitive performance.** (**A**) Scheme of the acquisition and reversal phase of the water maze test created with Biorender.com. (**B**) Escape latency during acquisition and reversal phases of the test, two-way ANOVA followed by Tukey’s multiple comparison test. Acquisition phase: Control (Ctrl) versus P2 *P* < 0.0001; Ctrl versus P30 *P* = 0.0003; P2 versus P60 *P* < 0.0001; P30 versus P60 *P* = 0.004. Reversal phase: Ctrl versus P2 *P* < 0.0001; P2 versus P30 *P* < 0.0001; P2 versus P60 *P* < 0.0001. (**C**) Velocity during acquisition and reversal phases of the test, two-way ANOVA followed by Tukey’s multiple comparison test. Acquisition phase: Ctrl versus P2 *P* < 0.0001; Ctrl versus P30 *P* < 0.0001; P2 versus P60 *P* = 0.0003; P30 versus P60 *P* = 0.004. Reversal phase: Ctrl versus P2 *P* < 0.0001; Ctrl versus P30 *P* < 0.0001; Ctrl versus P60 *P* < 0.0001. (**D**) Percentage of mice classified according to their predominant search strategy of the platform during the acquisition phase (two-way ANOVA followed by Tukey’s multiple comparison). Wall hugging (WH): Ctrl versus P2 ****P < 0.0001; Ctrl versus P30 ****P < 0.0001; P2 versus P30 ***P = 0.0005; P2 versus P60 ****P < 0.0001. Scanning (SC): Ctrl versus P2 ***P = 0.0004. Focal searching (FC): Ctrl versus P2 *P = 0.02. Direct swims (SP): P2 versus P60 *P = 0.04 and (**E**) reversal phase. Wall hugging (WH): Ctrl versus P2 ****P < 0.0001; Ctrl versus P30 ****P < 0.0001; P2 versus P60 ****P < 0.0001; P30 versus P60 ***P = 0.0005. Direct swims (SP): Ctrl versus P2 **P = 0.005; P2 versus P60 *P = 0.04). Data are shown as mean ± standard error of the mean (SEM), with circles representing individual mice. Ctrl *n* = 32, P2 *n* = 17, P30 *n* = 15, P60 *n* = 10. CH = chaining; CR = circling; FL = floating; RN = random swimming.

Finally, to test cognitive functions, we exploited the Morris water maze test. In this test, during the acquisition phase, mice had to learn the position of a hidden platform using spatial cues, while in the reversal phase, the position of the platform was changed and animals needed to learn the new location (Fig. 7A). P2-induced mice showed an increased escape latency compared to control mice in both the acquisition and reversal phases. Interestingly, P30-induced mice displayed a behaviour similar to P2- in the acquisition but a significant improvement in the reversal phase, where their escape latency curve overlapped with the controls (Fig. 7B). On the same line, the escape latency of P60-induced mice was comparable to control mice in both the acquisition and the reversal phases (Fig. 7B). This improvement occurred although the swimming velocity of P30- and P60-induced mice was slower compared to control mice, in both the acquisition and reversal phases for P30 and only in reversal phase for P60 (Fig. 7C). The same trend was observed for the escape latency, with P30-induced mice behaving like control animals only in the reversal phase and P60-induced mice in both phases, was evident also in other parameters determined including total distance (pathway), path efficiency, time spent in the target quadrant of the pool and time spent in the wall zone (Supplementary Fig. 9C–G). In the probe trial (first trial after the platform reversal), P60-induced mice spent even more time in the new target quadrant (OO) in comparison to control animals (Supplementary Fig. 9G), supporting that they were faster in relearning where the platform was placed. When looking at the strategies that mice adopt to reach the platform (Fig. 7D), the wall hugging strategy was more exploited by P2-induced mice compared to the P30- and P60- in the acquisition phase, that instead adopted more frequently the direct swim, in particular in the reversal phase (Fig. 7E).

Taken together, these results reveal that delayed induction of *Scn1a* gene haploinsufficiency elicits an intermediate score of performance in the cognitive ability of P30-induced mice and levels comparable to control mice in the P60-induced group.

## Discussion

In the present work, we generated a new murine model in which *Scn1a* gene haploinsufficiency can be induced in a temporally regulated manner. Exploiting this model, we ubiquitously induced the expression of a specific loss-of-function mutation in *Scn1a* gene (A1783V)^16^ at different stages of postnatal development, obtaining important information regarding the requirement of Na_v_1.1 physiological expression levels during and even after the critical time-window of neuronal circuit establishment at the early post-developmental stage. The phenotype of mice with *Scn1a* haploinsufficiency induced at P30 and P60 was compared to mice P2-induced, that developed a complete Dravet phenotype. In fact, whereas *Scn1a* gene expression starts postnatally and Na_v_1.1 protein is detected around P10–P12,^20^ we injected tamoxifen at P2–P7, likely achieving induction of *Scn1a* gene mutations before it was expressed or at least before its expression peaked.

Na_v_1.1 is mainly expressed in GABAergic interneurons with higher levels in PV subtypes^19,23,27^ and its haploinsufficiency is well known to induce a dramatic, although transient, impairment in their excitability.^19,28^

Correct functionality of GABAergic interneurons is extremely relevant for the proper maturation of neuronal circuits in different brain compartments, including cerebral cortex and hippocampus.^29-31^ In the cerebral cortex, in the first postnatal weeks, glutamatergic neurons increase their firing rates significantly and cortical activity is dominated by spontaneous waves of activity.^32-35^ Later during adolescence, the major inhibitory interneuron subtypes develop including PV, somatostatin (SST) and calretinin (CR) and inhibitory neurotransmission becomes more prominent, helping to refine the dynamics of pyramidal cell activity.^29,30^ Thus, the balance of excitatory to inhibitory inputs on pyramidal neurons progresses towards higher levels of inhibition.^36^ Similarly, in the development of cortico-hippocampal circuit, in the first postnatal weeks we assist to a progressive sparsification of dentate gyrus neuron firing, together with an increased temporal precision of granule cell activation.^37,38^ Both events are mainly mediated by changes in local circuit inhibition.^38^

In addition, alterations in the GABA excitatory/inhibitory shift^39^ have been described in Dravet syndrome models^40^ suggesting that a developmental delay in the maturation of GABAergic signalling may contribute to epileptogenesis in this pathology.

Considering the prominent role of GABAergic interneurons in the maturation of brain circuits, one can expect that granting their physiological activity at least during the brain circuit maturation in a Dravet context, would be sufficient to prevent symptom onset or at least to ameliorate them.

Interestingly, our data point out that this is not the case. In fact, delayed induction of *Scn1a* gene haploinsufficiency was not sufficient to impede the onset of spontaneous seizures, that appeared in both P30- and P60-induced mice. In P60-induced mice, we could even detect seizure onset that was between 10 days and 28 days since tamoxifen injections. In all the P60-induced analysed mice, high seizure frequency was observed in the first week after the onset, that is reminiscent of the traditional onset period of classic Dravet mice. Due to technical limitations, namely the necessity to achieve a minimal weight for mice to stand wireless transmitter implantations, we could not record the febrile stage in P2- and P30-induced mice, but only the chronic phase. Therefore, the increase in the frequency that is evident between P2-/P30- and P60-induced mice is mainly because we are observing them in different phases of the pathology. Indeed, in P60-induced mice, a drastic reduction in seizure frequency is observed in the following weeks, corresponding to their chronic phase. Comparison among the survival curves of the three experimental groups shows a small delay in the onset of SUDEP—and therefore of Dravet phenotype—in P60-induced mice, while the curves of P2- and P30-induced mice are perfectly overlapping. This may be due to an increased stability/decreased turnover of Na_v_1.1 in adult mice (almost P90 when symptoms start) in comparison to P2- and P30-induced mice that are P20 and P40 at symptom onset, respectively. This would implicate the requirement for a longer time before the system senses Na_v_1.1 haploinsufficiency. Alternatively, circuit consolidation is still incomplete at P30, while the process is fully established by P60, when circuits become more resistant to the development of Dravet syndrome upon Na_v_1.1 haploinsufficiency induction.

In light of these results, Na_v_1.1 appear to be a molecular switch for seizures, that start to appear as soon as the expression of the functional channel is halved. This observation is also corroborated by our previous complementary study, in which reactivation of *Scn1a* gene in a Dravet genetic background was sufficient to rescue both spontaneous and hyperthermia-induced seizures in juvenile mice at P30 and in adult mice at P90.^10^ Restated, *Scn1a* physiological expression is necessary and sufficient for proper brain activity regardless of the developmental stage as its delayed inactivation induces seizures while its re-expression in a Dravet genetic background rescues the seizure phenotype.

In Dravet mice with heterozygous *Scn1a* gene knock-out, GABAergic interneuron dysfunction is the trigger of seizures.^19-23^ The A1783V mutation alters Na_v_1.1 channel activation and slows down its inactivation, mostly maintaining unaltered the peak density of sodium current.^24^ Nevertheless, interneurons with this mutation have a firing impairment comparable to those carrying a complete loss-of-function.^24^ Similarly, hypoexcitable interneurons (both FS and non-FS), with an impairment in AP firing in comparison to age-matched controls, were observed in P2-induced mice. Although delayed induction of the expression of a sodium channel with abnormal electrophysiological properties could be different than activation of heterozygous deletion, a similar impact on interneuron firing was observed when the expression of the mutant Na_v_1.1 was induced at later stages, specifically at P30 and P60.

Given that in the *Scn1a^floxedA1783V/+^* model, mutation induction is Cre-dependent, to distinguish between FS and non-FS interneurons, we could not exploit interneuron-specific transgenic mouse lines (i.e. PV-Cre, SST-Cre, VIP-Cre) crossed with Ai9 mice. Therefore, we distinguished them based on their firing pattern and localization. Our results showed that both interneuron types are affected by the induction of mutant Na_v_1.1 at the different time points. However, considering that FS neurons with impaired firing become more similar to non-FS, we cannot completely exclude that some misclassification might have occurred. Specifically, in the Dravet background, this could have led to the inclusion of FS interneurons with impaired excitability in the non-FS group, resulting in an underestimation of the difference observed in the firing pattern between control and induced condition. Vice versa, some non-FS interneurons could have been mistaken for FS interneurons, leading to an overestimation of such difference in the firing. Nevertheless, the recordings obtained from biocytin-filled neurons whose identity was confirmed by *post hoc* immunofluorescence in the P2 group further support a reduction in the firing of both FS and non-FS Dravet interneurons compared to the control.

Finally, we investigated the appearance of behavioural alterations in delayed induced *Scn1a* haploinsufficient mice. Hyperactivity of Dravet mice is already evident from the pre-epileptic stage.^41^ This observation supposes that circuits relevant for this specific task are established early during postnatal development and leads to the hypothesis that maintaining normal circuit activity during that period would be sufficient for preventing their appearance. We did not confirm this hypothesis as also mice in which *Scn1a* normal expression was maintained up to P30 and even P60 displayed hyperactivity once its haploinsufficiency was induced. In addition, both P30- and P60-induced mice showed an anxious behaviour. In the open-field, P60-induced mice appeared even more anxious than the P2- and P30-induced mice and this may be explained by the fact that this group displayed very frequent seizures in the testing period, generally triggering anxiety. Different from the findings of the first report, describing the phenotype of mice carrying the A1783V *Scn1a* allele,^16^ P2-induced mice showed impaired sociability and social memory and these alterations also appeared in P30- and P60-induced mice. The mismatch with the previous report may be related to the different Cre strains exploited for mutation induction (UBC-Cre ERT2 versus CMV-Cre) or to diversity of the test by which this specific function was interrogated. Different output was obtained when cognitive function was tested by the Morris water maze, with P30-induced mice showing a significant amelioration and with P60- mice displaying a complete rescue in comparison to Dravet mice, suggesting that maintaining physiological levels of *Scn1a* up to P30 and P60 could provide some amelioration in cognition, at least in terms of spatial learning. Another interpretation of those results could also be that P30- and P60-induced mice at the time of cognitive testing (3–3.5 months) have experienced less time in seizures compared to P2-induced mice. An influential research standpoint has considered recurrent seizures as causative factor of cognitive decline.^42,43^ However, this aspect is still under debate as more recent evidence suggested that the cognitive course may be independent from epilepsy as disruptions in GABAergic firing may directly contribute to the poor cognitive outcomes in children with Dravet syndrome.^44-46^

The results of this study also have implications for precision medicine approaches that are being developed for this syndrome. Delayed induction of *Scn1a* gene haploinsufficiency cannot fully recapitulate the condition of Dravet patients receiving a gene therapy after diagnosis that is then discontinued, given that mice do not experience *Scn1a* haploinsufficiency perinatally, while patients do. However, it still can model the condition of an optimal gene therapy restoring normal levels of Na_v_1.1 administered transiently at birth in patients, diagnosed by a pre-natal/early postnatal screening. Indeed, ASOs have limited stability as their level progressively decreases^8^ and multiple doses are required throughout a patient’s life to boost Na_v_1.1 expression. Similarly, treatments based on artificial transcription factors boosting the transcription of *Scn1a* gene would require constitutive expression.^7,9^ Is the administration of such therapies required during the whole life of patients? What happens if due to some unexpected side effects, treatments need to be discontinued? In this study, we provide the first evidence that seizures and behavioural alterations will appear as soon as Na_v_1.1 haploinsufficiency is established. On one side, this suggests that protecting the patients during a critical development period might induce amelioration of cognitive tasks, at least in comparison to same age patients that did not receive that kind of treatment. On the other hand, each relapse toward gene haploinsufficiency could return to an acute phase of the pathology.

In conclusion, our findings uncover that most of Dravet syndrome phenotypic manifestations are not attributable to an interaction between Na_v_1.1 haploinsufficiency and development of brain circuits, but, conversely, strictly to the reduction of Na_v_1.1 function, as they also appear in an already established mature network. Our study also has implications for upcoming clinical trials in which *Scn1a* gene expression is reactivated and suggest that a transient *Scn1a* reinstatement during the critical window of postnatal brain development is not sufficient to prevent most adverse Dravet phenotypes, but it exerts a positive impact on cognitive function. However, sustained *Scn1a* expression throughout adulthood is needed to adequately sustain brain functions and, consequently, therapeutic protocols should be designed for continuous *Scn1a* expression to prevent impoverishment of the clinical benefits.

## Supplementary Material

awad350_Supplementary_Data

## Data Availability

The data that support the findings of this study are available from the corresponding author, upon reasonable request.
