# Supplementary material for: Temporal manipulation of the Scn1a gene reveals its essential role in adult brain function
Source: Brain. 2023 Oct 10;147(4):1216–30. doi: 10.1093/brain/awad350 (PMC10994529; doi:10.1093/brain/awad350)
Supplement: awad350_Supplementary_Data [file awad350_supplementary_data.zip › brain-2023-00982-File009.pdf]

Supplementary Materials

Temporal manipulation of *Scn1a* gene reveals its essential role in adult brain function

Claudia Di Berardino[1,†], Martina Mainardi[1,†], Simone Brusco[1,2†,#], Elena Benvenuto[1,3], Vania Broccoli[1,2] and Gaia Colasante[1]

Author affiliations:

1 Stem Cell and Neurogenesis Unit, Division of Neuroscience, IRCCS San Raffaele Scientific Institute, 20132 Milan, Italy

2 National Research Council (CNR), Institute of Neuroscience, 20129 Milan, Italy;

3 Gene and Cell Therapy PhD Program, Vita- Salute San Raffaele University, 20132 Milan, Italy
*present affiliation Axxam spa, Electrophysiology Unit, 20091, Bresso, Milan, Italy

Correspondence to: Dr. Gaia Colasante
Full address: Stem Cells and Neurogenesis Unit, Division of Neuroscience, IRCCS San Raffaele Scientific Institute,Via Olgettina 58, 20132 Milan, Italy. Tel: +39 02 26435790.
E-mail: colasante.gaia@hsr.it

Supplementary Figure 1

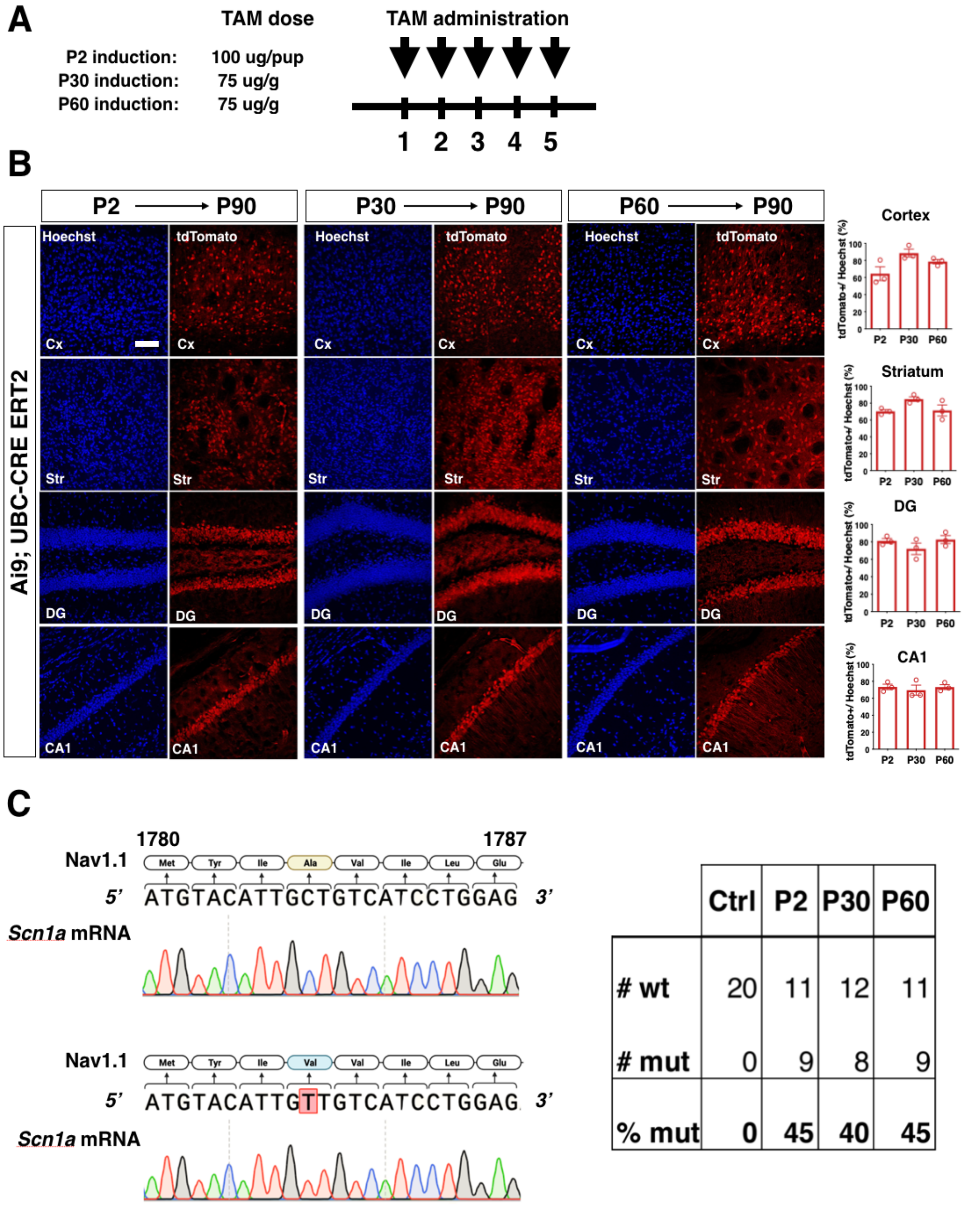

	Ctrl	P2	P30	P60
# wt	20	11	12	11
# mut	0	9	8	9
% mut	0	45	40	45

Supplementary Figure 1| Evaluation of efficiency of Cre-mediated recombination and mutation induction after tamoxifen administration in P2, P30 and P60 mice.
(**A**) Tamoxifen treatment schedule. (**B**) *Left,* representative images of immunofluorescence analysis for tdTomato in cortex (Cx), striatum (Str), dentate gyrus (DG) and CA1 of Ai9; UBC-Cre ERT2 mice treated with tamoxifen (*n*=3 for each timepoint). 40X, Scale bar = 20 µm. *Right*, percentage of TdTomato positive cells over Hoechst in each analyzed area. (**C**) Cortical tissues isolated from non-induced and P2-, P30-, and P60-induced *Scn1a*$^{floxedA1783V/+}$;UBC-Cre-ERT2 mice were processed from RNA extraction and Sanger sequencing of amplicons spanning the mutated region (exon 26). Sanger sequencing confirmed that transcript containing A1783V mutation is detectable in 45% of P2-induced mRNAs, 40% of P30-induced and 45% of P60-induced. Mutant mRNA was not detectable in samples from Ctrl mice.

Supplementary Figure 2

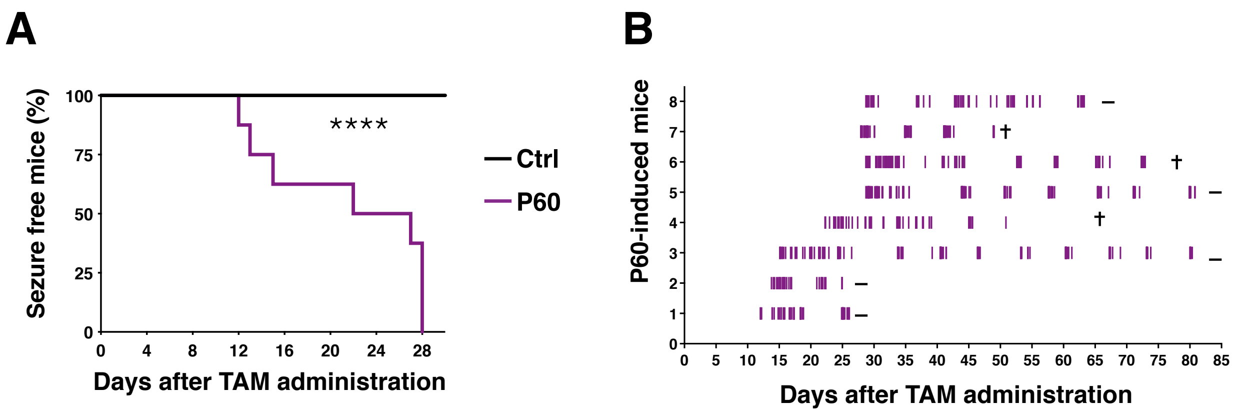

Supplementary Figure 2| Spontaneous seizures onset and occurrence in P60-induced mice. (**A**) Kaplan-Meier curve reporting the percentage of P60-induced mice subjected to video-EEG recording and developing spontaneous seizure after tamoxifen injection: 100% of mice developed the first spontaneous seizure by day 12 post treatment ($p<0.0001$, Log-rank test, $n = 8$). (**B**) Raster plot showing all generalized tonic-clonic seizures (Racine scale stages 4 and 5) in P60-induced mice ($n=8$) subjected to long term video-EEG monitoring. SUDEP event during recordings is indicated by a cross. Horizontal line indicates the end of video-EEG recording.

Supplementary Figure 3

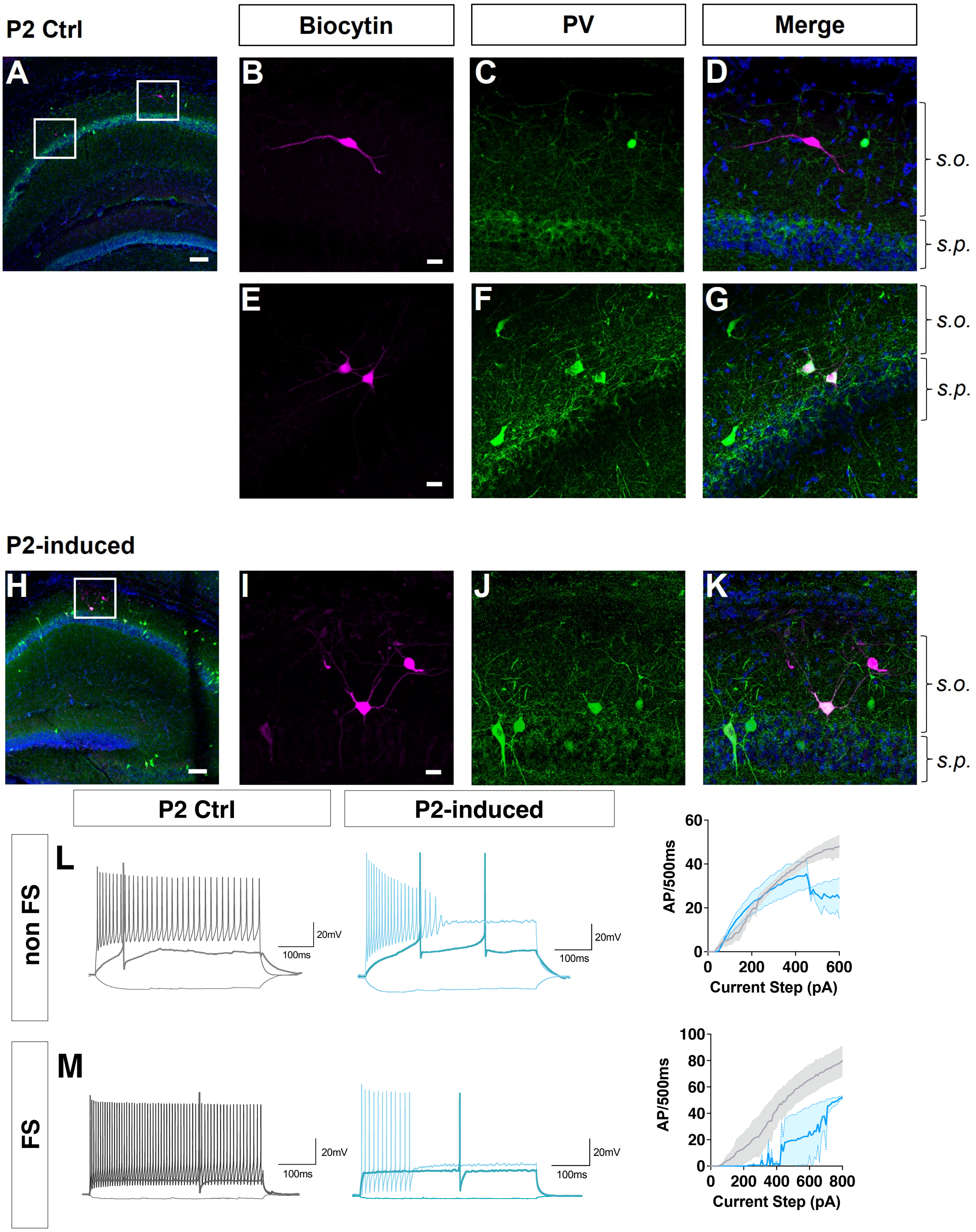

Supplementary Figure 3| *Post-hoc* immunostaining of biocytin-filled FS and non-FS interneurons in CA1 of P2 Ctrl and P2-induced mice following whole-cell patch clamp

recording. (**A-G**) Representative images of the immunostaining of biocytin-filled INs recorded in the CA1 of P2 Ctrl mice. (**A**) Image of the CA1 at low magnification (10X, scale bar = 100µm), showing 2 FS INs and 1 non-FS IN recorded in the same slice. White squares identify the cells-containing regions, imaged at higher magnification in (**B-G**). (**B-D**) Images showing the immunostaining for biocytin (**B**), PV (**C**) and merge (**D**) of one non-FS PV-negative IN in the *stratum oriens* (*s.o.*) at high magnification (40X, scale bar = 20µm). (**E-G**) Images showing the immunostaining for biocytin (**E**), PV (**F**) and merge (**G**) of two FS PV-positive INs at the border between *s.o.* and *stratum pyramidale* (*s.p.*) at high magnification (40X, scale bar = 20µm). (**H-K**) Representative images of the immunostaining of biocytin-filled INs recorded in the CA1 of P2-induced mice. (**H**) Image of the CA1 at low magnification (10X, scale bar = 100µm), showing 1 FS and 1 non-FS IN recorded in the same slice. White square identifies the cells-containing region, imaged at higher magnification in (**I-K**). (**I-K**) Images showing the immunostaining for biocytin (**I**), PV (**J**) and merge (**K**) of one FS PV-positive IN at the *s.o.-s-p.* border and a non-FS PV-negative IN in the *s.o.* at high magnification (40X, scale bar = 20µm). (**L**) Representative traces of biocytin-filled non-FS INs in CA1 of P2 Ctrl (left) and P2-induced mice (center) recorded at P18-P25. The voltage responses to one hyperpolarizing current step (-50pA) and two depolarizing steps (rheobase, thicker trace, and +500pA) are shown. *Right*, IO curve of the average firing rates in response to increasing current steps for non-FS PV-INs of the two experimental groups (n=5c/1a P2 Ctrl and 2c/1a P2-induced). **M**) Representative traces of biocytin-filled FS INs in CA1 of P2 Ctrl (left) and P2-induced mice (center) recorded at P18-P25. The voltage responses to one hyperpolarizing current step (-50pA) and two depolarizing steps (rheobase, thicker trace, and +700pA) are shown. *Right*, IO curve of the average firing rates in response to increasing current steps for FS PV+ INs of the two experimental groups (n=3c/1a P2 Ctrl and 2c/1a P2-induced).

Supplementary Figure 4

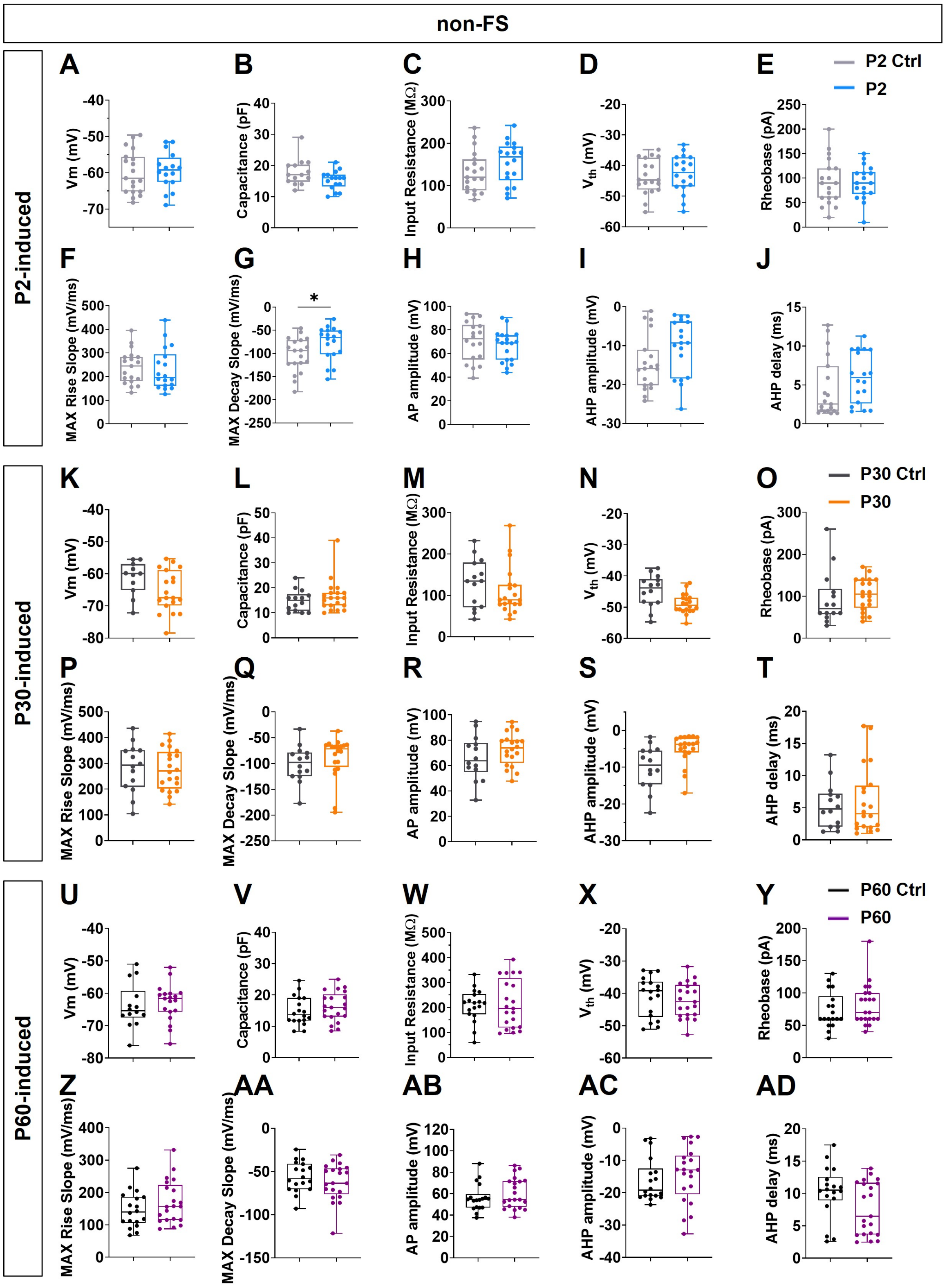

Supplementary Figure 4 | Properties of non-FS interneurons from Ctrl, P2-, P30- and P60-induced mice (A-J) Electrophysiological parameters of non-FS interneurons in CA1 of P2 Ctrl and

P2-induced mice recorded at P18-P25. Data distribution of neuronal passive membrane properties (**A-C**), firing threshold parameters (**D** and **E**) and action potential waveform (**F-J**) is reported. (**K-T**) Electrophysiological parameters of non-FS interneurons in CA1 of P30 Ctrl and P30-induced mice recorded at P45-P60. Data distribution of neuronal passive membrane properties (**K-M**), firing threshold parameters (**N** and **O**) and action potential waveform (**P-T**) is reported. (**U-AD**) Electrophysiological parameters of non-FS interneurons in CA1 of P60 Ctrl and P60-induced mice recorded at P85-P105. Data distribution of neuronal passive membrane properties (**U-Y**), firing threshold parameters (**X** and **Y**) and action potential waveform (**Z-AD**) is reported. Box plots display the median (internal horizontal line), first and third quartiles (upper and lower box edges) and minimal and max values (whiskers) of the data distribution. Circles represent individual data points from each cell. All parameters were analyzed by fitting data to a linear mixed model (LMM) using the Reduced Maximal Likelihood (REML) method followed by Sidak's *post-hoc* comparison. *n*, mean,SEM and *p* values are reported in Supplementary Table 1.

Supplementary Figure 5

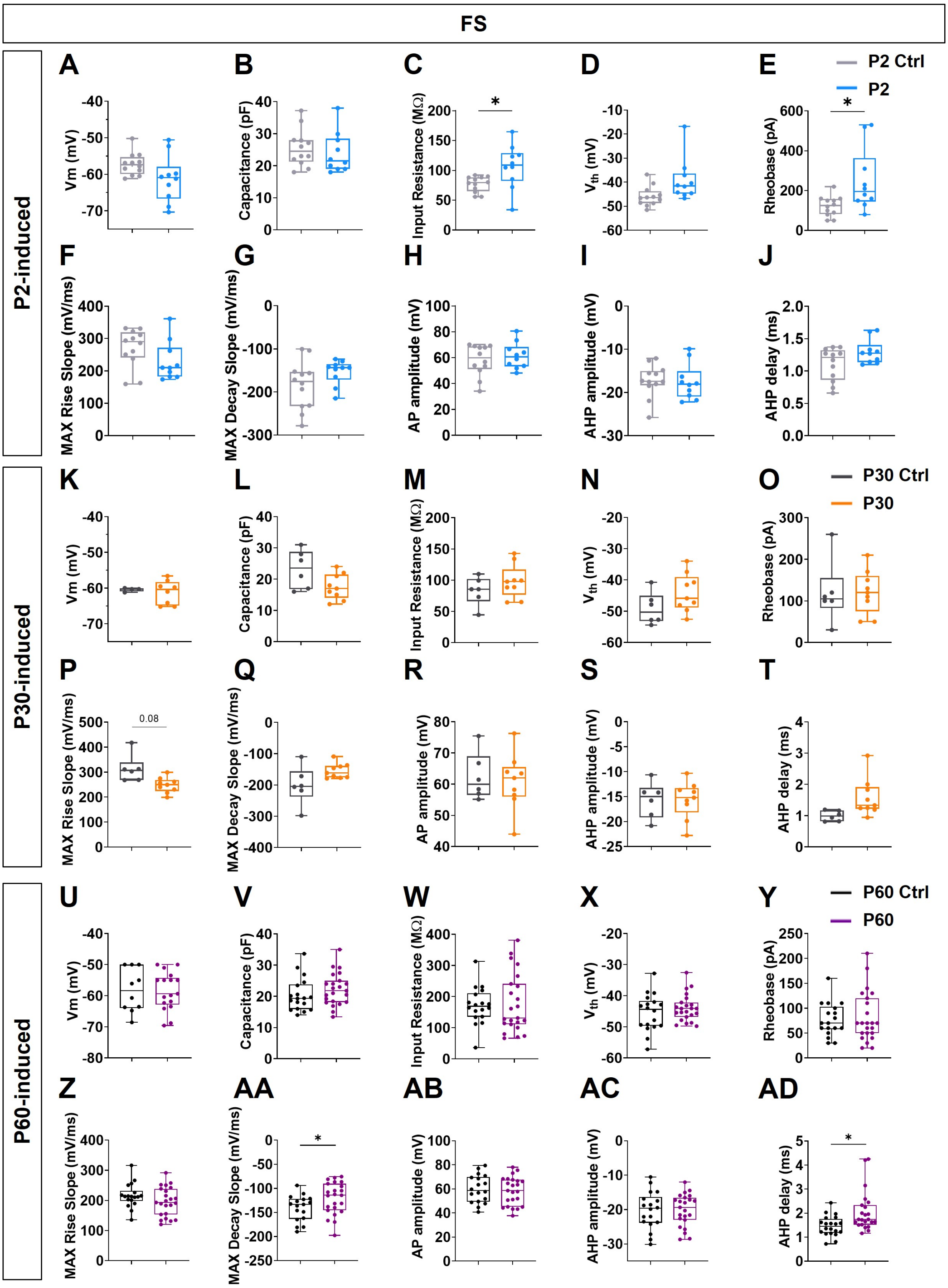

Supplementary Figure 5 | Properties of FS interneurons from Ctrl, P2-, P30- and P60-induced mice. (**A-J**) Electrophysiological parameters FS interneurons in CA1 of P2 Ctrl and P2-induced mice recorded at P18-P25. Data distribution of neuronal passive membrane properties (**A-C**), firing threshold parameters (**D** and **E**) and action potential waveform (**F-J**) is reported. (**K-T**) Electrophysiological parameters of FS interneurons in CA1 of P30 Ctrl and P30-induced mice recorded at P45-P60. Data distribution of neuronal passive membrane properties (**K-M**), firing threshold parameters (**N** and **O**) and action potential waveform (**P-T**) is reported. (**U-AD**) Electrophysiological parameters of FS interneurons in CA1 of P60 Ctrl and P60-induced mice recorded at P85-P105. Data distribution of neuronal passive membrane properties (**U-Y**), firing threshold parameters (**X** and **Y**) and action potential waveform (**Z-AD**) is reported. Box plots display the median (internal horizontal line), first and third quartiles (upper and lower box edges) and minimal and max values (whiskers) of the data distribution. Circles represent individual data points from each cell. All parameters were analyzed by fitting data to a linear mixed model (LMM) using the Reduced Maximal Likelihood (REML) method followed by Sidak's *post-hoc* comparison. *n*, mean, SEM and *p* values are reported in Supplementary Table 2.

Supplementary Figure 6

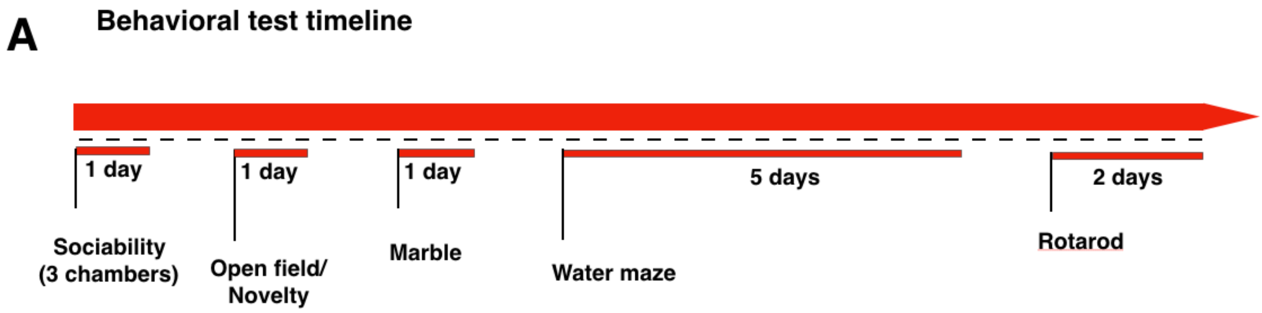

Supplementary Figure 6| Behavioral test timeline. (**A**) Sequence of execution of behavioral tests and relative duration of each test.

Supplementary Figure 7

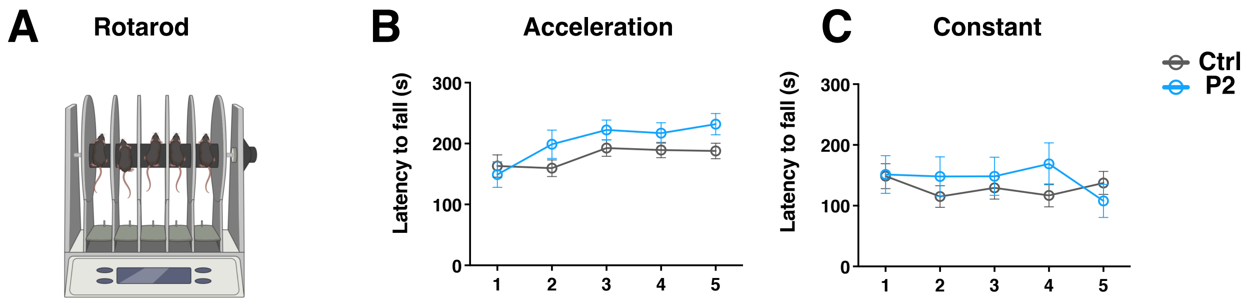

Supplementary Figure 7| Rotarod test in P2-induced mice and controls. (**A**) Scheme of the rotarod test created with Biorender.com. Latency to fall from the accelerating (**B**) or constant (**C**) rotarod (accelerating: *p* =0.24; constant: *p* =0.07; two-way ANOVA; Ctrl *n*=31; P2 *n*=12).

Supplementary Figure 8

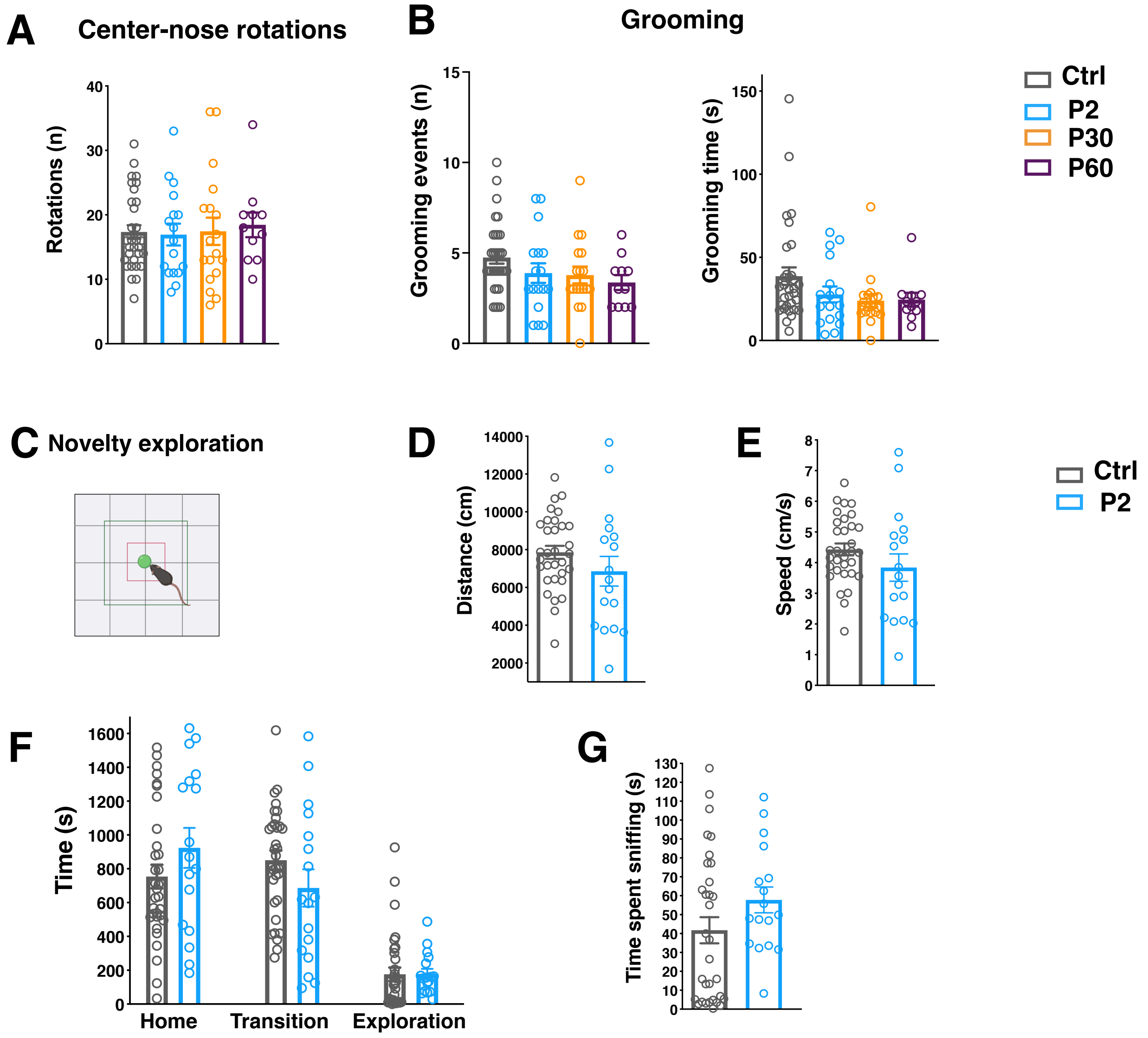

Supplementary Figure 8| Repetitive behaviors and novelty test. P2-, P30-, and P60-induced mice show levels of repetitive behaviors comparable to Ctrl mice.
(**A**) Number of center-nose rotations. (**B**) Total number of grooming events (*left*) and total time spent grooming (*right*). All the events were quantified during the first 10 minutes of the open field. (Ctrl *n*=32, P2 *n*=17, P30 *n*=18, P60 *n*=11). No statistical difference was reported between experimental groups (Kruskal-Wallis test followed by Dunn's multiple comparison test). Data are shown as mean ± SEM, with dots representing individual mice. (**C**) Scheme of novelty test, created with Biorender.com. P2 and Ctrl mice are reported. (**D**) Distance. (**E**) Velocity. (**F**) Time spent in home, transition and exploration areas after that a novel object has been placed in the center of the arena. (**G**) Time spent sniffing the novel object. (Ctrl *n*=32; P2 *n*=17), **D-G**, no statistical difference between the experimental group, t test. Data are shown as mean ± SEM; dots represent individual mice.

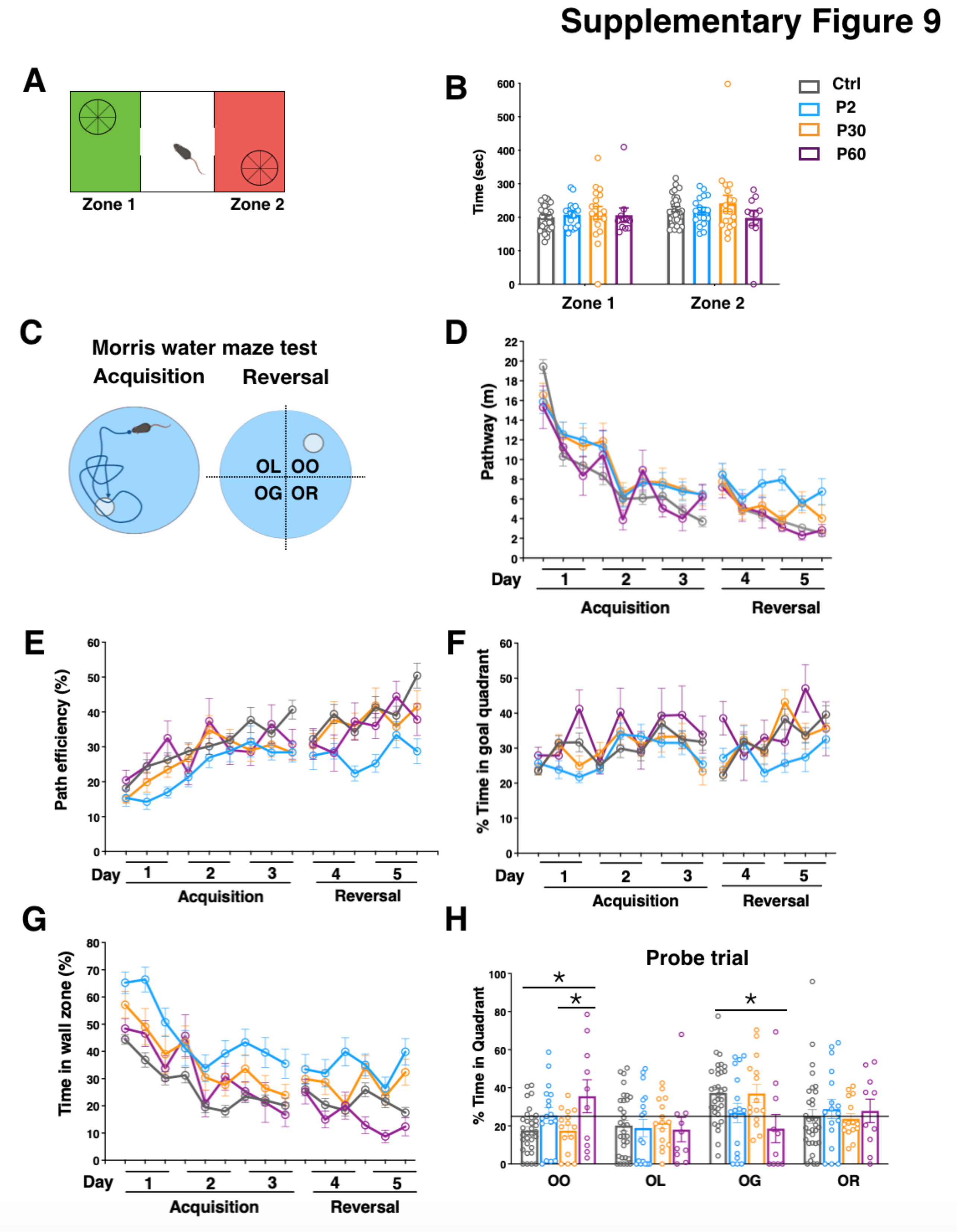

Supplementary Figure 9| Three-chamber and Morris water maze test in P2-, P30- and P60-induced mice. (**A**) Scheme of the habituation in the three-chamber test created with Biorender.com and (**B**) time spent in Zone 1 and Zone 2 by all the experimental groups. (**C**) Scheme of the Morris water maze test with abbreviations of each quadrant name: OG = Old Goal; OL= Old Left; OO= Opposite Old; OR= Opposite Right. (**D**) Total distance (Pathway) travelled during acquisition and

reversal phase, two-way ANOVA followed by Tukey's multiple comparison test (Acquisition phase: Ctrl vs P2 p=0.048; Ctrl vs P30 p= 0.0251. Reversal phase: Ctrl vs P2 p< 0.0001; P2 vs P30 p= 0.0090; P2 vs P60 p< 0.0001). (**E**) Path efficiency during acquisition and reversal phases of water maze test, two-way ANOVA followed by Tukey's multiple comparison test (Acquisition phase: Ctrl vs P2 p< 0.0001; Ctrl vs P30 p= 0.039; P2 vs P60 p= 0.006. Reversal phase: Ctrl vs P2 p< 0.0001; P2 vs P30 p= 0.0001; P2 vs P60 p= 0.007). (**F**) Percent of time spent in goal quadrant during acquisition and reversal phases of water maze test, two-way ANOVA followed by Tukey's multiple comparison test (Acquisition phase: P2 vs P60 p= 0.004. Reversal phase: Ctrl vs P2 p= 0.04; P2 vs P60 p= 0.005). (**G**) Percent of time spent in wall zone during acquisition and reversal phases of water maze test, two-way ANOVA followed by Tukey's multiple comparison test (Acquisition phase: Ctrl vs P2- p< 0.0001; Ctrl vs P30- p< 0.0001; P2- vs P30- p< 0.0001; P2- vs P60- p< 0.0001. Reversal phase: Ctrl vs P2- p< 0.0001; Ctrl vs P30- p= 0.004; Ctrl vs P60- p= 0.046; P2- vs P30- p= 0.025; P2- vs P60- p< 0.0001; P30- vs P60- p< 0.0001. Ctrl n=32, P2- n=17, P30- n=15, P60- n=10). (**H**) Percentage of time spent in each quadrant in the probe trial (19th trial, first after platform reversal), two-way ANOVA followed by Tukey's multiple comparison test (OO: Ctrl vs P60 p=0.025; OG: Ctrl vs P60 p= 0.0175; P30 vs P60 p= 0.0497).
