# Supplementary material for: Temporal manipulation of the Scn1a gene reveals its essential role in adult brain function
Source: Brain. 2023 Oct 10;147(4):1216–30. doi: 10.1093/brain/awad350 (PMC10994529; doi:10.1093/brain/awad350)
Supplement: awad350_Supplementary_Data [file awad350_supplementary_data.zip › brain-2023-00982-File010.pdf]

Supplementary Table 1. Properties of non-FS interneurons from Ctrl, P2-, P30- and P60-induced mice

**Non-FS**	**Ctrl**	**Scn1a haploinsufficiency induction**	**p value**
		**P2-induced**	
n (cells/animals)	19c/4a	18c/4a	
Cm (pF)	18.92 ± 1.05	15.75 ± 0.79	0.179
Ri (Mohm)	132.20 ± 11.06	154.60 ± 11.59	0.285
Vm (mV) (19c/4a;16c/4a)	-59.90 ± 1.35	-59.43 ± 1.28	0.805
Vth (mV)	-43.75 ± 1.31	-42.70 ± 1.45	0.478
Rheobase (pA)	92.11 ± 10.78	90.56 ± 8.22	0.924
Ith density (pA/pF)	5.30 ± 0.83	6.31 ± 0.82	0.545
MAX AP/500 ms	50.58 ± 2.94	37.15 ± 3.79	0.017 *
AP amplitude (mV)	66.70 ± 3.01	70.31 ± 3.99	0.796
AHP amplitude (mV)	-14.56 ± 1.62	-10.34 ± 1.73	0.434
MAX Rising slope (mV/ms)	240.10 ± 16.31	230.6 ± 20.85	0.626
MAX Decay slope (mV/ms)	-103.30 ± 8.62	-77.70 ± 8.60	0.043 *
AHP delay (ms)	4.51 ± 0.88	5.97 ± 0.77	0.517
Inflection rate	17.14 ± 1.27	16.84 ± 0.85	0.768
		**P30-induced**	
n (cells/animals)	14c/4a	20c/4a	
Cm (pF)	14.86 ± 1.13	16.65 ± 1.43	0.473
Ri (Mohm)	130.00 ± 15.61	109.70 ± 12.83	0.464
Vm (mV) (11c/4a;19c/4a)	-61.34 ± 1.60	-65.48 ± 1.49	0.160
Vth (mV)	-44.85 ± 1.39	-48.91 ± 0.70	0.063
Rheobase (pA)	94.29 ± 17.09	104.50 ± 8.66	0.566
Ith density (pA/pF)	6.20 ± 0.77	6.54 ± 0.53	0.717
MAX AP/500 ms	57.57 ± 6.60	35.45 ± 4.15	0.047 *
AP amplitude (mV)	66.04 ± 4.66	72.87 ± 2.89	0.309
AHP amplitude (mV)	-10.01 ± 1.58	-5.15 ± 0.91	0.168
MAX Rising slope (mV/ms)	282.70 ± 25.19	275.90 ± 17.84	0.841
MAX Decay slope (mV/ms)	-100.80 ± 9.45	-88.59 ± 9.04	0.629
AHP delay (ms)	5.35 ± 0.94	6.06 ± 1.17	0.962
Inflection rate	16.04 ± 1.48	20.33 ± 0.34	0.024 *
		**P60-induced**	
n (cells/animals)	18c/7a	21c/8a	
Cm (pF)	14.80 ± 1.08	16.27 ± 1.03	0.839
Ri (Mohm)	208.00 ± 15.36	213.90 ± 21.09	0.75
Vm (mV) (11c/5a;17c/8a)	-66.80 ± 1.19	-64.04 ± 1.15	0.166
Vth (mV)	-40.98 ± 1.46	-42.23 ± 1.18	0.503
Rheobase (pA)	73.33 ± 6.67	81.90 ± 6.99	0.726
Ith density (pA/pF)	5.21 ± 0.47	5.64 ± 0.74	0.632
MAX AP/500 ms	37.06 ± 2.23	29.05 ± 1.93	0.020 *
AP amplitude (mV)	56.13 ± 3.00	60.59 ± 3.14	0.35
AHP amplitude (mV)	-16.12 ± 1.58	-14.42 ± 1.89	0.522
MAX Rising slope (mV/ms)	146.30 ± 13.00	169.90 ± 14.40	0.236
MAX Decay slope (mV/ms)	-56.05 ± 4.15	-62.23 ± 4.65	0.335
AHP delay (ms)	10.20 ± 0.95	7.67 ± 0.89	0.06
Inflection rate	14.43 ± 0.53	15.44 ± 0.52	0.303

Values are reported as mean ± sem. N (cells/animals) is indicated for each experimental group. * p < 0,05.
For resting membrane potential (Vm) n is indicated when different, due to exclusion of cells displaying spontaneous firing at rest.

Supplementary Table 2. Properties of FS interneurons from Ctrl, P2-, P30- and P60-induced mice

FS	Ctrl	Scn1a haploinsufficiency induction	P value
		**P2-induced**	
n (cells/animals)	12c/5a	10c/4a	
Cm (pF)	25.52 ± 1.67	23.99 ± 2.01	0.845
Ri (Mohm)	76.99 ± 3.74	106.60 ± 11.53	0.048 *
Vm (mV)	-57.27 ± 0.88	-61.29 ± 2.01	0.117
Vth (mV)	-45.87 ± 1.18	-39.23 ± 2.77	0.085
Rheobase (pA)	121.70 ± 14.50	250.00 ± 49.80	0.015 *
Ith density (pA/pF)	4.88 ± 0.61	9.90 ± 1.38	0.002 **
MAX AP/500 ms	96.83 ± 8.30	64.50 ± 5.76	0.047 *
AP amplitude (mV)	58.10 ± 3.45	61.48 ± 3.20	0.496
AHP amplitude (mV)	-17.28 ± 1.09	-17.55 ± 1.30	0.728
MAX Rising slope (mV/ms)	270.50 ± 17.17	229.30 ± 19.08	0.456
MAX Decay slope (mV/ms)	-184.40 ± 16.15	-153.70 ± 9.13	0.133
AHP delay (ms)	1.11 ± 0.07	1.30 ± 0.06	0.131
Inflection rate	16.76 ± 9.96	16.62 ± 1.33	0.985
		**P30-induced**	
n (cells/animals)	6c/3a	9c/4a	
Cm (pF)	23.17 ± 2.50	17.56 ± 1.37	0.098
Ri (Mohm)	82.93 ± 9.28	97.82 ± 8.91	0.284
Vm (mV) (4c/3a;8c/3a)	-60.45 ± 0.26	-61.17 ± 1.18	0.684
Vth (mV)	-49.17 ± 2.11	-44.14 ± 2.03	0.120
Rheobase (pA)	120.00 ± 30.88	121.10 ± 17.44	0.673
Ith density (pA/pF)	5.19 ± 1.22	6.95 ± 0.86	0.245
MAX AP/500 ms	109.83 ± 9.41	78.00 ± 7.68	0.012 *
AP amplitude (mV)	62.36 ± 3.10	60.75 ± 2.97	0.723
AHP amplitude (mV)	-15.70 ± 1.48	-15.67 ± 1.25	0.988
MAX Rising slope (mV/ms)	313.40 ± 22.39	247.60 ± 10.07	0.087
MAX Decay slope (mV/ms)	-201.10 ± 25.09	-155.20 ± 7.90	0.187
AHP delay (ms)	0.99 ± 0.07	1.58 ± 0.20	0.144
Inflection rate	19.63 ± 1.10	19.63 ± 1.79	0.999
		**P60-induced**	
n (cells/animals)	18c/8a	23c/7a	
Cm (pF)	20.43 ± 1.25	22.22 ± 1.07	0.402
Ri (Mohm)	171.20 ± 13.90	176.40 ± 19.59	0.796
Vm (mV) (10c/6a;17c/7a)	-58.24 ± 2.19	-58.49 ± 1.48	0.907
Vth (mV)	-45.49 ± 1.41	-43.96 ± 0.89	0.346
Rheobase (pA)	76.67 ± 7.84	81.74 ± 10.42	0.775
Ith density (pA/pF)	3.85 ± 0.42	3.81 ± 0.51	0.994
MAX AP/500 ms	79.00 ± 6.63	51.65 ± 3.15	<0.0005 ***
AP amplitude (mV)	59.97 ± 2.70	58.30 ± 2.43	0.822
AHP amplitude (mV)	-20.28 ± 1.28	-20.12 ± 0.97	0.917
MAX Rising slope (mV/ms)	216.30 ± 9.36	195.80 ± 9.89	0.149
MAX Decay slope (mV/ms)	-140.90 ± 6.51	-120.60 ± 6.89	0.045 *
AHP delay (ms)	1.52 ± 0.09	2.05 ± 0.17	0.016 *
Inflection rate	13.33 ± 1.06	14.64 ± 0.74	0.445

Values are reported as mean ± sem. N is indicated for each experimental group. * p < 0,05; ** p < 0,005; *** p < 0,0005
For resting membrane potential (Vm) n is indicated when different, due to exclusion of cells displaying spontaneous firing at rest.
